# Unequal distribution of genetically-intact HIV-1 proviruses in cells expressing the immune checkpoint markers PD-1 and/or CTLA-4

**DOI:** 10.3389/fimmu.2023.1064346

**Published:** 2023-01-26

**Authors:** Katie Fisher, Timothy E. Schlub, Zoe Boyer, Thomas A. Rasmussen, Ajantha Rhodes, Rebecca Hoh, Frederick M. Hecht, Steven G. Deeks, Sharon R. Lewin, Sarah Palmer

**Affiliations:** ^1^ Centre for Virus Research, The Westmead Institute of Medical Research, The University of Sydney, Sydney, NSW, Australia; ^2^ Sydney Medical School, Westmead Clinical School, Faculty of Medicine and Health, The University of Sydney, Sydney, NSW, Australia; ^3^ Sydney School of Public Health, Faculty of Medicine and Health, The University of Sydney, Sydney, NSW, Australia; ^4^ Department of Infectious Diseases, The University of Melbourne at The Doherty Institute for Infection and Immunity, Melbourne, VIC, Australia; ^5^ Department of Infectious Diseases, Aarhus University Hospital, Aarhus, Denmark; ^6^ Division of HIV, Infectious Diseases and Global Medicine, Department of Medicine, Zuckerberg San Francisco General Hospital, University of California San Francisco, San Francisco, CA, United States; ^7^ Department of Infectious Diseases, Alfred Hospital and Monash University, Melbourne, VIC, Australia; ^8^ Victorian Infectious Diseases Service, Royal Melbourne Hospital at The Doherty Institute for Infection and Immunity, Melbourne, VIC, Australia

**Keywords:** HIV-1, proviruses, persistence, PD-1, CTLA-4, genetically-intact

## Abstract

**Introduction:**

HIV-1 persists in resting CD4^+^ T-cells despite antiretroviral therapy (ART). Determining the cell surface markers that enrich for genetically-intact HIV-1 genomes is vital in developing targeted curative strategies. Previous studies have found that HIV-1 proviral DNA is enriched in CD4^+^ T-cells expressing the immune checkpoint markers programmed cell death protein-1 (PD-1) or cytotoxic T-lymphocyte associated protein-4 (CTLA-4). There has also been some success in blocking these markers in an effort to reverse HIV-1 latency. However, it remains unclear whether cells expressing PD-1 and/or CTLA-4 are enriched for genetically-intact, and potentially replication-competent, HIV-1 genomes.

**Methods:**

We obtained peripheral blood from 16 HIV-1-infected participants, and paired lymph node from four of these participants, during effective ART. Memory CD4^+^ T-cells from either site were sorted into four populations: PD-1^-^CTLA-4^-^ (double negative, DN), PD-1^+^CTLA-4^-^ (PD-1^+^), PD-1^-^CTLA-4^+^ (CTLA-4^+^) and PD-1^+^CTLA-4^+^ (double positive, DP). We performed an exploratory study using the full-length individual proviral sequencing (FLIPS) assay to identify genetically-intact and defective genomes from each subset, as well as HIV-1 genomes with specific intact open reading frames (ORFs).

**Results and Discussion:**

In peripheral blood, we observed that proviruses found within PD-1^+^ cells are more likely to have intact ORFs for genes such as *tat*, *rev* and *nef* compared to DN, CTLA-4^+^ and DP cells, all of which may contribute to HIV-1 persistence. Conversely, we observed that CTLA-4 expression is a marker for cells harbouring HIV-1 provirus that is more likely to be defective, containing low levels of these intact ORFs. In the lymph node, we found evidence that CTLA-4^+^ cells contain lower levels of HIV-1 provirus compared to the other cell subsets. Importantly, however, we observed significant participant variation in the enrichment of HIV-1 proviruses with intact genomes or specific intact ORFs across these memory CD4^+^ T-cell subsets, and therefore consideration of additional cellular markers will likely be needed to consistently identify cells harbouring latent, and potentially replication-competent, HIV-1.

## Introduction

Despite the success of antiretroviral therapy (ART) in suppressing HIV-1 replication and disease progression in HIV-1-infected individuals, the virus persists in resting CD4^+^ T-cells during effective therapy (1–4). These persistent proviruses are the major barrier to a cure for HIV-1, as the virus will rapidly rebound from these cells upon the interruption of therapy (5–7). Identification of cellular markers for CD4^+^ T-cells that contain persistent genetically-intact HIV-1 during therapy is necessary for the development of potential curative strategies. Many studies have identified specific CD4^+^ T-cell subsets, such as effector memory (EM), T helper 1 (Th1) or T follicular helper (Tfh) cells (8–11), or specific cellular markers, such as HLA-DR (12), as enriched for total HIV-1 DNA, genetically-intact HIV-1, or inducible replication-competent HIV-1 during ART. However, it is likely that there is considerable overlap between these cell types, making the identification of cellular markers or features involved in HIV-1 persistence difficult.

Immune checkpoint blockade (ICB) targeting the cell surface markers programmed cell death protein-1 (PD-1) and/or cytotoxic T lymphocyte-associated protein-4 (CTLA-4) has been identified as an effective strategy in the treatment of malignant cancers (13). Several studies have demonstrated effectiveness of anti-PD-1 or anti-CTLA-4 treatment in reactivating latent HIV-1 *in vitro* or *ex vivo* (14, 15). A range of single case reports and small case series of HIV-1-infected individuals on ART with malignant cancers receiving either anti-PD-1 or anti-CTLA-4 or both *in vivo* reported reactivation of the latent HIV-1 reservoir, though there have been some mixed results (16–21). Recently, virological sub-studies of two prospective clinical trials of HIV-1-infected individuals on ART of more than 30 participants clearly demonstrated that latency reversal occurs *in vivo* following a single dose of anti-PD-1 (pembrolizumab) (22) and following a combination of anti-PD-1 (nivolumab) and anti-CTLA-4 (ipilimumab), with enhanced responses after multiple doses of antibody (23). Studies of SIV-infected macaques have similarly shown some ability of these treatments to reactivate latent SIV within ART-treated macaques, with combination blockade inducing enhanced effects, indicating a potential role of PD-1 and CTLA-4 in SIV and HIV-1 persistence (24).

Previous studies have indicated that persistent HIV-1 may be enriched in CD4^+^ T-cells expressing PD-1 or CTLA-4 in both the peripheral blood and in lymphoid organs. For example, PD-1 has been shown to be a marker for the enrichment of total HIV-1 DNA in both treated and untreated individuals (15, 25). Moreover, peripheral blood CM and TM cells that express PD-1 were found to be significantly enriched in HIV-1 DNA compared to CM and TM cells that do not express PD-1 (25). The co-expression of PD-1 with other immune checkpoint markers, TIGIT and LAG-3, on peripheral blood CD4^+^ T-cells was shown to significantly enrich for integrated HIV-1 DNA compared to expression of single markers (26). Translation-competent HIV-1 has also been shown to be enriched in peripheral blood PD-1^+^ cells (27), and infected cells containing transcription and translation-competent provirus were enriched in cells co-expressing PD-1, CTLA-4 and TIGIT in untreated viremic participants (28). Peripheral blood CD4^+^ T-cells expressing high levels of CTLA-4 during untreated HIV-1 infection were shown to be enriched for HIV-1 DNA compared to those expressing lower levels of CTLA-4 (29). In the lymph node, Tfh cells, which express PD-1 and CXCR5, have been shown to be an important reservoir for replication-competent HIV-1 in both viremic and ART-suppressed HIV-1-infected individuals (8, 9). In addition, a study of SIV-infected macaques indicated enrichment of SIV DNA in CTLA-4^+^ PD-1^-^ CD4^+^ T-cells in the lymph node (30). In a recent study from our team, we demonstrated a modest enrichment of HIV-1 DNA in cells expressing both PD-1 and CTLA-4 compared to double negative cells in the peripheral blood, but that the provirus within these cells was more difficult to activate in these double positive cells compared to double negative cells (31).

However, no study to date has specifically investigated the distribution of genetically-intact and defective proviruses in cells that express immune checkpoint markers in both the peripheral blood and tissue. The current study therefore extends the findings of Rasmussen et al. (2022) (31) to investigate the composition of HIV-1 proviruses within a subset of these participants. Using the full-length individual proviral sequencing (FLIPS) assay, we perform an exploratory study to investigate the distribution of genetically-intact and defective HIV-1 proviruses in memory CD4^+^ T-cells expressing PD-1 and/or CTLA-4 from peripheral blood and paired lymph node samples from a subset of these participants, with the aim of identifying areas for further investigation.

## Materials and methods

### Study participants and design

Participants were recruited from a prospective observational study of HIV-1-infected individuals on ART who had blood and lymph node collected, and findings from this study were recently reported (31). In brief, a total of 16 participants treated with suppressive ART for at least 3 years were included in the current sub-study, 11 of which were enrolled at The Alfred Hospital, Melbourne, Australia, and 5 of which were enrolled at the University of California San Francisco (UCSF), California, USA. Leukapheresis samples were obtained from all 16 participants. Additionally, excisional lymph node biopsies were available for 4 participants. Fifteen of the participants were infected with HIV-1 subtype B, while one participant was infected with HIV-1 subtype CRF01_AE. These participants were chosen from the original cohort (n=21) (31) on the basis that they had sufficient cells available in storage to allow for sequencing of the provirus and the predicted size of the overall proviral reservoir. Clinical details of the participants in this study and the parent study are provided in **Supplementary Table 1**, with the participant samples included in this study bolded.

Ethics approval for the study was obtained from the institutional review board at the Western Sydney Local Health District, which includes the Westmead Institute for Medical Research, the Human Research Ethics Committees at The Alfred and Avenue Hospitals in Melbourne, the University of Melbourne Ethics Committee, and the Institutional Review Board at UCSF. All participants provided written informed consent.

### Sample processing and cell sorting

Leukapheresis and lymph node biopsy samples were processed, and cell sorting was performed as described in Rasmussen et al. (2022) (31). Briefly, for peripheral blood mononuclear cells (PBMCs) obtained by leukapheresis, memory CD4^+^ T-cells were first isolated by negative selection using the MojoSort Human CD4 memory T cell isolation kit (BioLegend, San Diego, CA, USA, Cat no 480064), followed by sorting. Lymph node mononuclear cells (LNMCs) were isolated from the lymph node biopsies, followed by sorting. The four peripheral blood and lymph node cell subsets obtained for the study following sorting were: double negative (CD3^+^CD4^+^CD45RA^-^PD-1^-^CTLA-4^-^; DN), PD-1^+^CTLA-4^-^ (CD3^+^CD4^+^CD45RA^-^PD-1^+^CTLA-4^-^; PD-1^+^), PD-1^-^CTLA-4^+^ (CD3^+^CD4^+^CD45RA^-^PD-1^-^CTLA-4^+^; CTLA-4^+^), and double positive (CD3^+^CD4^+^CD45RA^-^PD-1^+^CTLA-4^+^; DP). Representative plots for the sorting strategy can be found in **Supplementary Figure 1**, and in Rasmussen et al. (2022) (31). As shown in Rasmussen et al. (2022), to avoid collecting cells that overlap between the four cell populations, small gaps between the individual gates for each cell population were introduced during cell sorting (31). The purity of the sorted populations was also assessed on a subset of samples. The median purity was 90.1% (IQR 76.95-97.3%) (31).

### DNA extraction and full-length individual proviral sequencing

As described in Rasmussen et al. (2022) (31), sorted cells were lysed using Buffer RLT (RNeasy Lysis Buffer; Qiagen) and stored at -80°C prior to extraction. Cell-associated DNA was extracted from the lysates using the Qiagen All-prep kit (Qiagen, Hilden, Germany).

Near-full-length proviral DNA amplicons were obtained from the extracted DNA for each cell subset using the full-length individual proviral sequencing (FLIPS) assay, as described in Hiener et al. (2017) (10, 32). Briefly, the extracted DNA was diluted to limiting dilution, followed by two rounds of a nested PCR targeting the 5’ and 3’ LTRs to amplify near-full-length proviral genomes. Extracted DNA from the 15 participants infected with HIV-1 subtype B was amplified using the subtype B-specific primers described in Hiener et al. (2017) (10). Extracted DNA from the single participant infected with HIV-1 subtype CRF01_AE was amplified as described in Wang et al. (2022) (33). Reagent concentrations and cycling conditions were unchanged for these primers. Amplicons are then prepared for next generation sequencing on the Illumina MiSeq platform (10).

Following sequencing, HIV-1 contigs were assembled using one of two assembly methods. The first method used a previously-designed custom workflow in CLC Genomics (Qiagen) (10). The second method is a custom pipeline for contig *de novo* assembly (34, 35), and can be found at https://github.com/jsede/virus_assembly. Briefly, the generated reads are QC trimmed using BBDuk v 37.98 (https://sourceforge.net/projects/bbmap/). MEGAHIT v1.1.3 (36) was then used to *de novo* assemble draft genomes, followed by mapping the reads onto the draft genome for confirmation using BBMap v37.98. This confirmation is visualised using Geneious Prime v.2020.0.3. The final majority consensus genome is then extracted, and this is used for further analysis.

### Sequence analysis

Generated proviral genomes were classified as genetically-intact or defective (10, 37). Briefly, genomes were first scanned for regions containing inversions, followed by large internal deletions of size >100bp. The remaining genomes of size >8800bp were scanned for frameshift mutations, premature stop codons or incorrect start codons within HIV-1 open reading frames (ORFs) using the Los Alamos database tool GeneCutter (https://www.hiv.lanl.gov/content/sequence/GENE_CUTTER/cutter.html) and the National Cancer Institute (NCI) Proviral Sequence Database (PSD) Proviral Sequence Annotation & Intactness Test (https://psd.cancer.gov/tools/tool_index.php) (38). Hypermutated sequences were identified using the Los Alamos tool Hypermut (https://www.hiv.lanl.gov/content/sequence/HYPERMUT/hypermut.html) (39). Any remaining genomes were scanned for defects in the packaging signal or major splice donor (MSD) site (position 744-745) (10, 40). Genomes with a mutated MSD were still classified as genetically-intact if they contained a cryptic splice donor site at HXB2 position 748-749 (10, 37, 41). The remaining genomes were classified as genetically-intact.

Genomes with genetically-intact *p24*, *tat*, *rev*, rev response element (RRE) or *nef* ORFs were classified using GeneCutter and the NCI PSD Proviral Sequence Annotation & Intactness Test (https://psd.cancer.gov/tools/tool_index.php) (38). Groups of two or more sequences within the same cell subset that were identified as 100% identical to one another were classified as an expansion of identical sequences (EIS). Genetically-identical sequences were identified using the Los Alamos database tool ElimDupes (https://www.hiv.lanl.gov/content/sequence/elimdupesv2/elimdupes.html). Tropism of proviral genomes were identified using the tool Web PSSM (https://indra.mullins.microbiol.washington.edu/webpssm/).

### Statistics

Comparisons of infection frequency or the proportion of proviruses with a particular characteristic between cellular subsets were made using logistic regression (**Supplementary Table 2**). The correlated nature of this dataset (repeated observations/cells for each participant) was accounted for by using a mixed logistic regression with a random effect for the intercept and grouped by participant. When convergence or boundary issues arose with this analysis, the random effect for intercept, grouped by participant, was replaced with a fixed effect for participant. This is indicated in **Table 1** and **Supplementary Table 2** by p-values with a “*”. Effect modification was additionally investigated by testing the inclusion of an interaction term between participant and cellular subset in the fixed effect only (no random effect) model and is shown in the final column of **Table 1** and **Supplementary Table 2**. To further reduce issues associated with model convergence and complex effect modification between participants and cellular subsets, comparisons were made in a pairwise fashion rather than a single regression with all subsets included.

**Supplementary Table 2** shows all comparison results across the four subsets (six pairwise comparisons) within two anatomic locations (peripheral blood and lymph node) for several different but not independent outcomes (overall infection frequency, intact infection frequency, proportion intact *p24*, etc). This results in 312 statistical comparisons. Importantly, these 312 comparisons are not independent to one another. For example, trends in infection frequency may match trends in intact infection frequency, or trends in peripheral blood may match trends in lymph nodes. Therefore, the ideal adjustment for multiple comparisons is unclear. As this is exploratory work, we address this with a false discovery rate calculation that estimates what proportion of comparisons below some p-value cut-off threshold (*a*) are likely to be spurious, calculated as


$$\text{False discovery rate}=\frac{n_{a}}{aN}$$

Where *n_a_* is the number of observed p-values below the threshold *a* and *N* is the total number of p-values calculated (312 in **Supplementary Table 2**). Two columns of p-values (no effect modification, and participant effect modification) are treated as a single comparison, and if either of these p-values are below the threshold it will contribute to *n_a_*.This false discovery rate will be artificially high due to the non-independence of comparisons, and so represents an upper bound (worst-case scenario) of spurious results. Due to this lack of independence of comparisons we also encourage readers to interpret p-values as a scale of the level of statistical evidence – with smaller p-values indicating stronger evidence and a lower possibility of spurious findings – as opposed to a categorical significant/non-significant based on a significance threshold (e.g. p=0.05). This is in alignment with modern practice of interpretation of statistical results (42).

Logistic regressions were conducted in R (43). Mixed logistic regressions were conducted using R library glmer (44). Spearman’s correlation tests were conducted using Prism software (GraphPad).

### Data availability

All sequences included in this manuscript have been deposited in GenBank (accession numbers OP700895-OP701628).

## Results

We used FLIPS (10) to sequence and genetically-characterise near-full-length HIV-1 proviral genomes from four subsets of resting CD4^+^ T-cells from the peripheral blood and from the lymph node, sorted based on their expression of PD-1 and/or CTLA-4: double negative (DN), PD-1^+^CTLA-4^-^ (PD-1^+^), PD-1^-^CTLA-4^+^ (CTLA-4^+^) and double positive (DP) (**Table 2**). We compared different properties of the HIV-1 proviruses found within each of these cell subsets, the full results of which are presented in **Supplementary Table 2**. Below we describe in further detail the results of a number of these comparisons that are most relevant to the persistence of HIV-1 proviruses, and more specifically genetically-intact proviruses, within memory CD4^+^ T-cell subsets that express PD-1 and/or CTLA-4 (**Table 1**). For these comparisons, we tested whether there was a significant difference in a particular property of HIV-1 proviruses between two cellular subsets from the enrolled participants (difference between subsets). In addition, we determined whether the magnitude or direction of these differences in properties of the HIV-1 provirus were affected by participant-specific variation (participant effect modification). Importantly, we stress that this is an exploratory study, with a level of possible false discovery (see Methods), where we have identified targets of further investigation in order to confirm the enrichment of HIV-1 proviruses within CD4^+^ T cells expressing PD-1 and/or CTLA-4.

**Table 1:** Calculated p-values indicating differences in specific properties of HIV-1 proviruses between subsets or participant effect modification.

Comparison	Measurement	Anatomic location	Subsets compared	Difference between subsets in this data (p-value)	Direction	Participant effect modification (p-value)
Cells HIV-1^+^ positive	Infection frequency per 10^6^ cells	PB	DN vs PD-1^+^	<0.00001	PD-1^+^>DN	<0.00001
			DN vs CTLA-4^+^	0.85		<0.00001
			DN vs DP	0.78		<0.00001
			PD-1^+^ vs CTLA-4^+^	<0.00001	PD-1^+^>CTLA-4^+^	<0.00001
			PD-1^+^ vs DP	0.0001	PD-1^+^>DP	<0.00001
			CTLA-4^+^ vs DP	0.86		<0.00001
		LN	DN vs PD-1^+^	0.61		0.2
			DN vs CTLA-4^+^	0.006	DN>CTLA-4^+^	0.05
			DN vs DP	0.19		<0.00001
			PD-1^+^ vs CTLA-4^+^	0.001	PD-1^+^>CTLA-4^+^	0.03
			PD-1^+^ vs DP	0.16*		<0.00001
			CTLA-4^+^ vs DP	0.0002	DP>CTLA-4^+^	0.6
Genetically-Intact	Infection frequency per 10^6^ cells	PB	DN vs PD-1^+^	0.72		0.009
			DN vs CTLA-4^+^	0.01	DN>CTLA-4^+^	0.34
			DN vs DP	0.03	DN>DP	0.3
			PD-1^+^ vs CTLA-4^+^	0.04	PD-1^+^>CTLA-4^+^	0.19
			PD-1^+^ vs DP	0.2		0.21
			CTLA-4^+^ vs DP	0.92*		0.98
		LN	DN vs PD-1^+^	0.06*		1
			DN vs CTLA-4^+^	1*		1
			DN vs DP	0.59*		1
			PD-1^+^ vs CTLA-4^+^	0.12*		1
			PD-1^+^ vs DP	0.13*		0.69
			CTLA-4^+^ vs DP	0.41*		1
Intact *p24* ORF	Infection frequency per 10^6^ cells	PB	DN vs PD-1^+^	<0.00001	PD-1^+^>DN	<0.00001
			DN vs CTLA-4^+^	0.29		0.06
			DN vs DP	0.33		0.0003
			PD-1^+^ vs CTLA-4^+^	<0.00001	PD-1^+^>CTLA-4^+^	<0.00001
			PD-1^+^ vs DP	<0.00001	PD-1^+^>DP	0.0005
			CTLA-4^+^ vs DP	0.88*		0.05
		LN	DN vs PD-1^+^	0.58		0.24
			DN vs CTLA-4^+^	0.28		0.14
			DN vs DP	0.53*		0.001
			PD-1^+^ vs CTLA-4^+^	0.09		0.28
			PD-1^+^ vs DP	0.39*		0.02
			CTLA-4^+^ vs DP	0.19		0.62
Intact *p24* ORF	Proportion proviruses	PB	DN vs PD-1^+^	0.11		0.01
			DN vs CTLA-4^+^	0.1		0.07
			DN vs DP	0.26		0.21
			PD-1^+^ vs CTLA-4^+^	0.0001	PD-1^+^>CTLA-4^+^	0.002
			PD-1^+^ vs DP	0.003	PD-1^+^>DP	0.2
			CTLA-4^+^ vs DP	0.65		0.69
Intact *nef* ORF	Infection frequency per 10^6^ cells	PB	DN vs PD-1^+^	<0.00001	PD-1^+^>DN	<0.00001
			DN vs CTLA-4^+^	0.002	DN>CTLA-4^+^	0.02
			DN vs DP	0.07		0.12
			PD-1^+^ vs CTLA-4^+^	<0.00001	PD-1^+^>CTLA-4^+^	0.0001
			PD-1^+^ vs DP	<0.00001	PD-1^+^>DP	0.01
			CTLA-4^+^ vs DP	0.25		0.16
		LN	DN vs PD-1^+^	0.23		0.18
			DN vs CTLA-4^+^	0.85		0.8
			DN vs DP	0.35*		0.64
			PD-1^+^ vs CTLA-4^+^	0.13*		0.04
			PD-1^+^ vs DP	0.006	PD-1^+^>DP	0.74
			CTLA-4^+^ vs DP	0.98*		0.52
Intact *nef* ORF	Proportion proviruses	PB	DN vs PD-1^+^	0.002	PD-1^+^>DN	0.007
			DN vs CTLA-4^+^	0.0006	DN>CTLA-4^+^	0.006
			DN vs DP	0.31		0.17
			PD-1^+^ vs CTLA-4^+^	<0.00001	PD-1^+^>CTLA-4^+^	0.004
			PD-1^+^ vs DP	0.0001	PD-1^+^>DP	0.43
			CTLA-4^+^ vs DP	0.03	DP>CTLA-4^+^	0.14
Intact tat/rev/RRE provirus	Infection frequency per 10^6^ cells	PB	DN vs PD-1^+^	<0.00001	PD-1^+^>DN	0.0001
			DN vs CTLA-4^+^	0.008*	DN>CTLA-4^+^	0.07
			DN vs DP	0.24*		0.09
			PD-1^+^ vs CTLA-4^+^	<0.00001	PD-1^+^>CTLA-4^+^	0.0005
			PD-1^+^ vs DP	<0.00001	PD-1^+^>DP	0.11
			CTLA-4^+^ vs DP	0.22		0.4
		LN	DN vs PD-1^+^	0.08*		0.08
			DN vs CTLA-4^+^	0.95		1
			DN vs DP	0.81*		0.83
			PD-1^+^ vs CTLA-4^+^	0.18*		0.05
			PD-1^+^ vs DP	0.01	PD-1^+^>DP	0.7
			CTLA-4^+^ vs DP	0.98*		0.52
Intact tat/rev/RRE provirus	Proportion proviruses	PB	DN vs PD-1^+^	0.0001	PD-1^+^>DN	0.02
			DN vs CTLA-4^+^	0.006	DN>CTLA-4^+^	0.03
			DN vs DP	0.46		0.26
			PD-1^+^ vs CTLA-4^+^	<0.00001	PD-1^+^>CTLA-4^+^	0.04
			PD-1^+^ vs DP	<0.00001	PD-1^+^>DP	0.69
			CTLA-4^+^ vs DP	0.03	DP>CTLA-4^+^	0.48
Sequences part of an EIS	Proportion proviruses	PB	DN vs PD-1^+^	0.001	PD-1^+^>DN	<0.00001
			DN vs CTLA-4^+^	0.24		0.0001
			DN vs DP	0.84		0.03
			PD-1^+^ vs CTLA-4^+^	0.02	PD-1^+^>CTLA-4^+^	<0.00001
			PD-1^+^ vs DP	0.049	PD-1^+^>DP	0.01
			CTLA-4^+^ vs DP	0.23		0.008
Proviruses with an inversion	Proportion	PB	DN vs PD-1^+^	0.2*		0.55
			DN vs CTLA-4^+^	0.83		0.89
			DN vs DP	0.09		0.3
			PD-1^+^ vs CTLA-4^+^	0.46		0.95
			PD-1^+^ vs DP	0.32		0.61
			CTLA-4^+^ vs DP	0.1		0.78
Proviruses with a deletion	Proportion	PB	DN vs PD-1^+^	0.03	DN>PD-1^+^	0.0002
			DN vs CTLA-4^+^	0.23		0.36
			DN vs DP	0.91		0.02
			PD-1^+^ vs CTLA-4^+^	0.0001	CTLA-4^+^>PD-1^+^	<0.00001
			PD-1^+^ vs DP	0.04	DP>PD-1^+^	0.008
			CTLA-4^+^ vs DP	0.33		0.19
Hypermutated proviruses	Proportion	PB	DN vs PD-1^+^	0.45*		0.39
			DN vs CTLA-4^+^	0.44		0.72
			DN vs DP	0.91		0.14
			PD-1^+^ vs CTLA-4^+^	0.14*		0.23
			PD-1^+^ vs DP	0.37		0.046
			CTLA-4^+^ vs DP	0.33		0.33
Proviruses with a premature stop codon in an ORF	Proportion	PB	DN vs PD-1^+^	0.6		0.97
			DN vs CTLA-4^+^	1*		1
			DN vs DP	1*		1
			PD-1^+^ vs CTLA-4^+^	0.04*	PD-1^+^>CTLA-4^+^	1
			PD-1^+^ vs DP	0.06*		1
			CTLA-4^+^ vs DP	1*		1
Proviruses with a frameshift mutation in an ORF	Proportion	PB	DN vs PD-1^+^	0.11		0.98
			DN vs CTLA-4^+^	0.16*		1
			DN vs DP	0.16*		1
			PD-1^+^ vs CTLA-4^+^	0.0001*		1
			PD-1^+^ vs DP	0.32*		1
			CTLA-4^+^ vs DP	1*		1
Proviruses with a *cis*-acting defect	Proportion	PB	DN vs PD-1^+^	<0.00001	PD-1^+^>DN	0.2
			DN vs CTLA-4^+^	0.08		0.99
			DN vs DP	0.98		0.97
			PD-1^+^ vs CTLA-4^+^	<0.00001	PD-1^+^>CTLA-4^+^	1
			PD-1^+^ vs DP	<0.00001	PD-1^+^>DP	0.99
			CTLA-4^+^ vs DP	0.14		1

* Random effect for intercept, grouped by participant, has been replaced by a fixed effect for participant due to convergence or boundary issues in the mixed effects logistic regression analysis.

**Table 2:** Numbers and characteristics of proviruses amplified per cell subset and participant.

PID	Anatomic location	Cell subset	Cells analysed	Defective	Intact *p24*	Intact *nef*	Intact tat/rev/RRE	Genetically-Intact
2208	Peripheral blood	DN	395,062	13	2	2	1	1
		PD-1^+^	395,062	12	4	4	4	1
		CTLA-4^+^	285,847	3	1	1	0	0
		DP	474,074	7	2	3	1	0
2651	Peripheral blood	DN	395,062	5	3	3	3	1
		PD-1^+^	369,506	22	18	24	17	8
3162	Peripheral blood	DN	422,654	20	12	5	2	0
		PD-1^+^	260,062	43	18	3	2	0
		CTLA-4^+^	28,834	1	0	0	0	0
		DP	67,165	1	0	0	0	0
3147	Peripheral blood	DN	395,062	6	2	2	1	1
		PD-1^+^	395,062	43	21	29	24	0
		CTLA-4^+^	464,869	20	0	0	0	0
		DP	137,101	1	0	0	0	0
5003	Peripheral blood	DN	379,475	3	3	3	2	2
		PD-1^+^	426,816	10	10	9	8	6
		CTLA-4^+^	395,062	3	0	0	0	0
		DP	149,383	1	1	0	0	0
PRA001	Peripheral blood	DN	517,265	26	15	11	10	5
		PD-1^+^	500,579	30	14	11	9	2
		CTLA-4^+^	191,400	5	4	0	0	0
		DP	121,037	4	2	0	0	0
PRA002	Peripheral blood	DN	927,034	7	4	3	2	2
		PD-1^+^	1,010,000	1	0	0	0	0
		CTLA-4^+^	884,896	1	0	0	0	0
		DP	1,010,000	3	0	1	0	0
PRA003	Peripheral blood	DN	1,464,815	10	8	7	7	4
		PD-1^+^	1,481,481	6	1	5	5	0
		CTLA-4^+^	1,121,914	5	2	3	3	1
		DP	1,414,815	4	2	2	1	1
	Lymph node	DN	500,000	14	6	1	0	0
		PD-1^+^	474,684	12	5	4	4	2
		CTLA-4^+^	154,034	0	0	0	0	0
		DP	640,000	1	0	0	0	0
PRA004	Peripheral blood	DN	518,519	20	5	3	1	1
		PD-1^+^	553,086	18	12	4	5	0
		CTLA-4^+^	336,661	7	2	0	0	0
		DP	622,222	27	8	2	1	0
PRA005	Peripheral blood	DN	518,519	22	13	9	8	2
		PD-1^+^	518,519	43	38	38	38	2
		CTLA-4^+^	622,222	19	10	4	4	0
		DP	563,457	22	10	11	10	0
PRA006	Peripheral blood	DN	1,455,334	10	7	3	3	0
		PD-1^+^	1,443,716	14	7	7	7	2
		CTLA-4^+^	618,519	15	3	0	0	0
		DP	1,467,130	23	9	7	6	2
	Lymph node	DN	200,000	0	0	0	0	0
		PD-1^+^	754,442	8	2	1	1	0
		DP	249,750	4	2	0	0	0
PRA007	Peripheral blood	DN	679,012	25	9	7	4	1
		PD-1^+^	679,012	7	1	3	2	0
		CTLA-4^+^	509,259	24	10	5	4	2
		DP	679,012	6	2	3	3	1
	Lymph node	DN	123,697	0	0	0	0	0
		PD-1^+^	783,784	12	6	6	5	2
		CTLA-4^+^	314,545	0	0	0	0	0
		DP	779,918	9	5	1	1	1
PRA008	Peripheral blood	DN	679,012	13	10	7	6	1
		PD-1^+^	679,012	18	10	5	5	0
		CTLA-4^+^	509,259	23	15	2	1	0
		DP	679,012	21	11	6	5	2
PRA009	Peripheral blood	DN	567,284	13	2	1	0	0
		PD-1^+^	419,753	34	11	3	2	2
		CTLA-4^+^	398,148	14	3	2	1	1
		DP	364,198	34	11	0	1	0
	Lymph node	DN	90,000	2	0	1	1	0
		PD-1^+^	195,000	3	3	0	0	0
		CTLA-4^+^	90,000	2	1	1	1	0
		DP	33,028	3	1	0	0	0
PRA010	Peripheral blood	DN	659,259	6	2	1	1	0
		PD-1^+^	659,259	11	4	2	1	0
		CTLA-4^+^	675,926	10	1	2	2	0
		DP	663,426	4	0	0	0	0
PRA011	Peripheral blood	DN	643,981	19	5	0	0	0
		PD-1^+^	647,901	26	14	7	6	0
		CTLA-4^+^	663,580	18	6	4	3	1
		DP	667,438	16	8	2	2	1

### Genetically-intact HIV-1 proviruses are less frequent in CD4^+^ T-cells expressing CTLA-4

**Figure 1:**
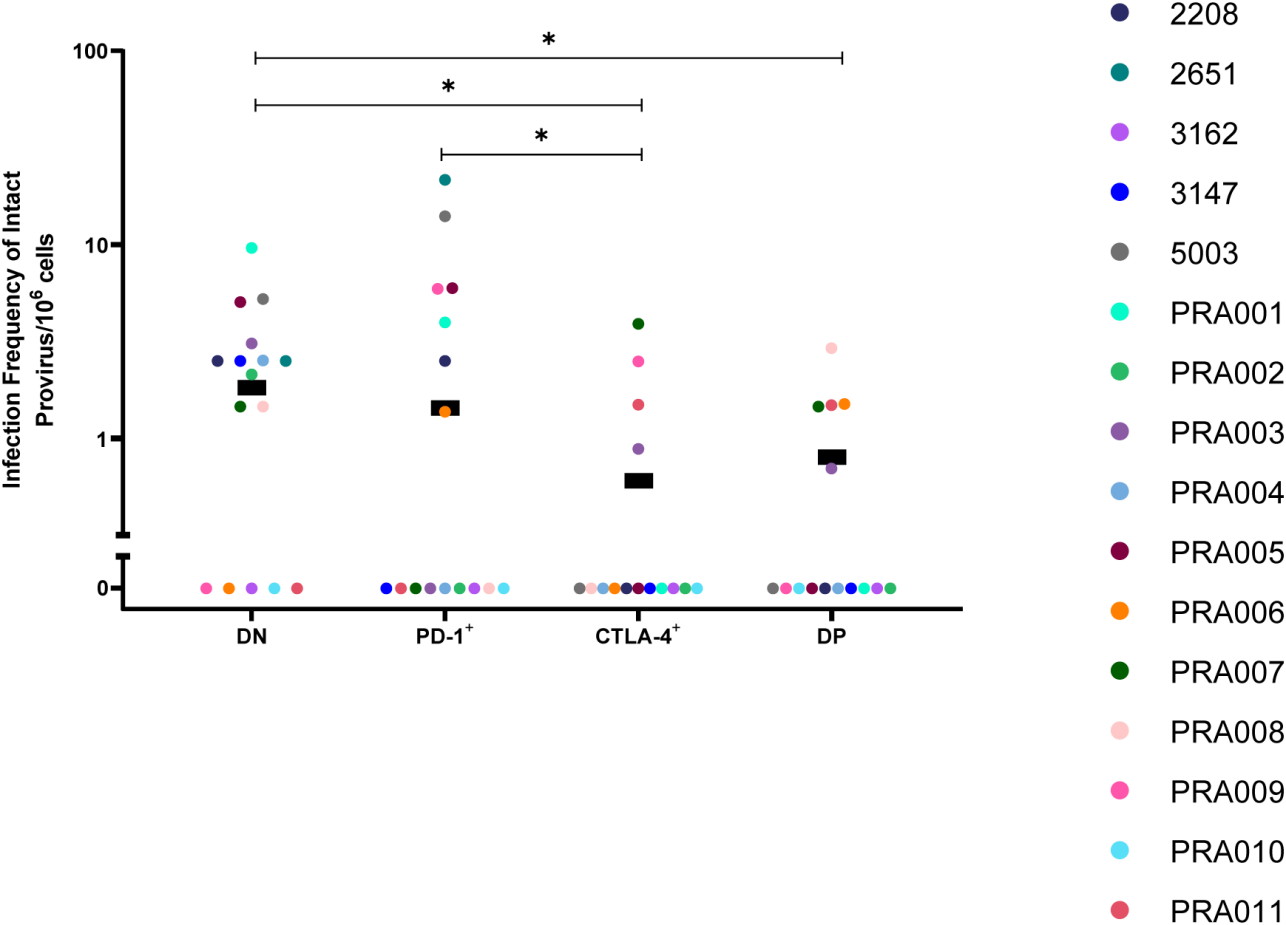
Genetically-intact HIV-1 proviruses in peripheral blood memory CD4^+^ T-cells expressing PD-1 and/or CTLA-4. The infection frequency per 10^6^ cells of genetically-intact HIV-1 proviruses in DN, PD-1^+^, CTLA-4^+^ and DP cells. Data is the estimated infection frequency per 10^6^ cells for each subset. p-values represent evidence for an overall trend of a difference between subsets within these participants (mixed effects logistic regression), * p ≤ 0.05.

It is well-known that the majority of HIV-1 proviruses found within CD4^+^ T-cells of HIV-1-infected individuals are genetically-defective, and therefore unlikely to be replication-competent (40, 45). We therefore first investigated the distribution of genetically-intact proviruses within peripheral blood cells sorted based on PD-1 and CTLA-4 expression. Consistent with this known abundance of genetically-defective proviral genomes in the cells of ART-suppressed individuals, we observed a low number of genetically-intact genomes across the individual participants, ranging from 0-8 genetically-intact genomes per participant cell subset (**Table 2**). We used the number of available cells per subset per participant, the total number of HIV-1 proviral genomes and the number of genetically-intact proviral genomes amplified from those cells (**Table 2**) to calculate the infection frequency per 10^6^ cells of genetically-intact HIV-1 proviruses. Overall, we observed that DN and PD-1^+^ cells had a higher estimated infection frequency of genetically-intact genomes compared to CTLA-4^+^ and DP cells (**Figure 1**). We found weak evidence for PD-1^+^ cells having a higher intact infection frequency compared to CTLA-4^+^ cells within this data (p=0.04; **Figure 1**). We also found evidence for DN cells having a higher intact infection frequency compared to CTLA-4^+^ cells (p=0.01) and DP cells (p=0.03) within this data (**Figure 1**). This indicates that the expression of CTLA-4 on resting CD4^+^ T-cells may be a marker for a lower infection frequency of genetically-intact proviruses. We did find evidence for a difference in the infection frequency of genetically-intact proviruses between DN and PD-1^+^ cells, but these differences were highly variable across different participants (participant effect modification p=0.009; **Table 1**). This may indicate a participant-specific role for PD-1 expression in enrichment for genetically-intact proviruses. We did not observe evidence for any participant-dependent differences between DP cells and PD-1^+^ cells or DP cells and CTLA-4^+^ cells (**Supplementary Table 2**).

### Proviruses that can express p24 protein are enriched in PD-1^+^ cells

As a surrogate for investigating replication-competency, Pardons et al. (2019) found that latently-infected cells from ART-suppressed HIV-1-infected individuals that are able to express HIV-1 p24 protein in response to stimulation were enriched in PD-1^+^ cells (27). However, recent studies have shown that genetically-defective proviruses are able to express the p24 protein, as well as other HIV-1 proteins (34, 46–50). We therefore investigated whether proviruses with particular genetic characteristics are enriched in CD4^+^ T-cells expressing PD-1 and/or CTLA-4 in order to determine whether PD-1^+^ cells are enriched for proviruses that have the capability to express specific HIV-1 proteins.

**Figure 2:**
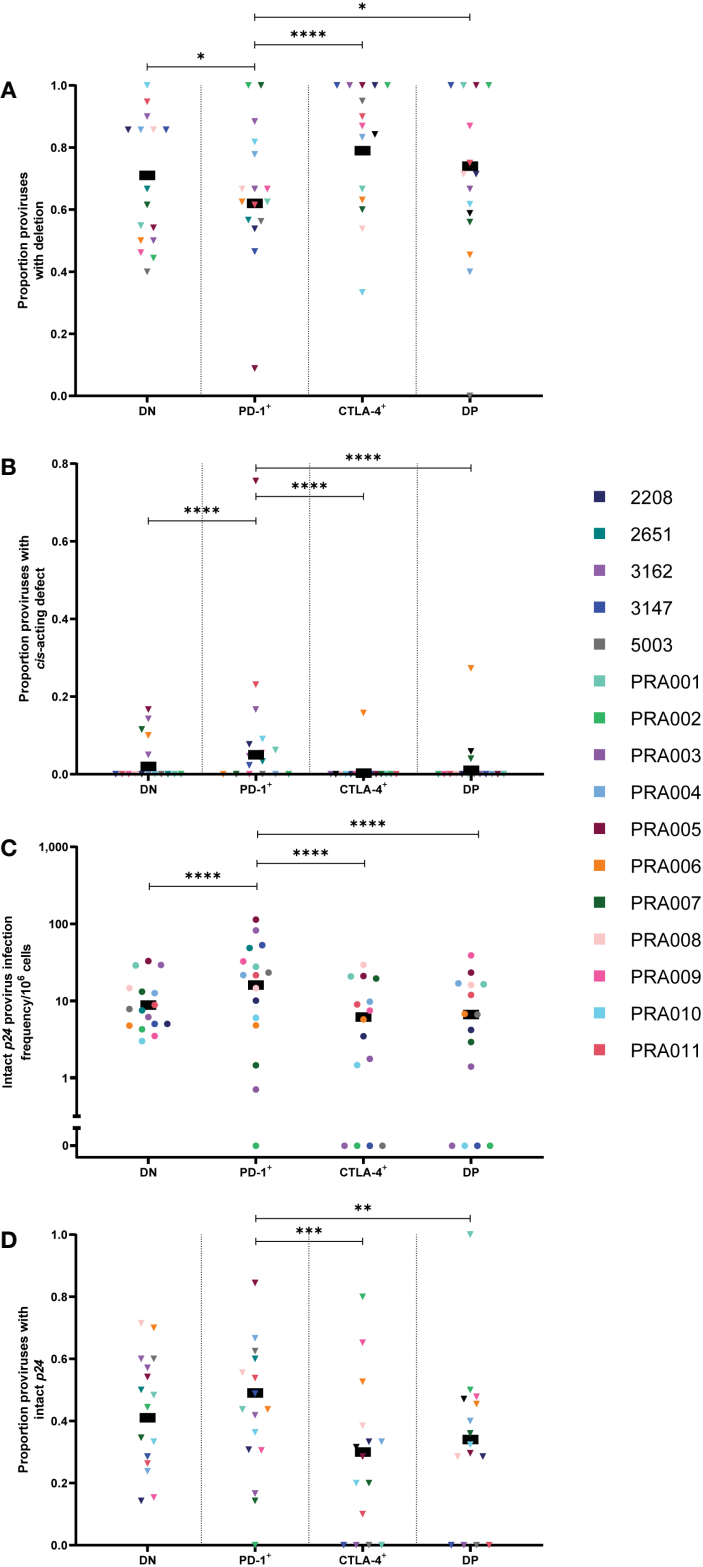
HIV-1 proviruses with an intact *p24* open reading frame in peripheral blood memory CD4^+^ T-cells expressing PD-1 and/or CTLA-4. The proportion of amplified proviruses that contained either a large internal deletion **(A)** or a mutation in the *cis*-acting region **(B)** from DN, PD-1^+^, CTLA-4^+^ and DP cells. The HIV-1 infection frequency per 10^6^ cells of proviruses with an intact *p24* ORF in DN, PD-1^+^, CTLA-4^+^ and DP cells **(C)**. The proportion of amplified proviruses that contained an intact *p24* ORF in DN, PD-1^+^, CTLA-4^+^ and DP cells **(D)**. Data is the estimated infection frequency per 10^6^ cells or proportion for each subset. p-values represent evidence for an overall trend of a difference between subsets within these participants (mixed effects logistic regression), * p ≤ 0.05, ** p ≤ 0.01, *** p ≤ 0.001, **** p ≤ 0.0001.

We first compared the proportion of cells containing certain types of genetic defects between the DN, PD-1^+^, CTLA-4^+^ and DP cell subsets. Note that the classification of proviruses as defective follows a process of elimination, with sequences classified as containing inversions, followed by large internal deletions, hypermutation, premature stop codons, frameshift mutations or defects in the *cis*-acting region (10, 37). This means that the classification of these defects is not mutually-exclusive. No significant differences were observed between the four cell subsets for inversions or hypermutation (**Table 1**). When comparing the proportion of proviruses with large internal deletions between the four peripheral blood cell subsets, we observed evidence that any differences between subsets, particularly between PD-1^+^ cells and each of the other cell subsets, were largely dependent on participant-specific variation (**Table 1**). Overall, however, for this data we observed that CTLA-4^+^ cells had the highest estimated proportion of proviruses with large internal deletions, followed by DP cells, DN cells and PD-1^+^ cells. We found strong evidence that the proportion of sequences carrying a large internal deletion was higher in CTLA-4^+^ cells than PD-1^+^ cells within this data (p=0.0001; **Figure 2A**) and evidence that PD-1^+^ cells had a lower proportion of proviruses with large internal deletions compared to DN cells (p=0.03) and DP cells (p=0.04) within this data (**Figure 2A**). However, we did find evidence that each of these differences varied across participants (participant effect modification; CTLA-4^+^ vs PD-1^+^ cells: p<0.00001, DN vs PD-1^+^ cells: p=0.0002, DP vs PD-1^+^ cells: p=0.008; **Table 1**). This observation that CTLA-4^+^ cells had the highest proportion of proviruses with a large internal deletion is consistent with the low infection frequency of genetically-intact proviruses observed in CTLA-4^+^ cells. Altogether, this indicates that PD-1^+^ cells are more likely to harbour genomes that are full-length, and therefore more likely to express HIV-1 proteins such as p24, compared to PD-1^-^ and CTLA-4^+^ cells.

Proviruses with defects in the *cis*-acting region encompass those with deletions in any of the four stem loops that form part of the packaging signal within the 5’-LTR of the HIV-1 provirus, or those with point mutations in the major splice donor (MSD) site (10, 40). In this study, these genomes are genetically-intact in all regions of the HIV-1 genome except for the *cis*-acting region. Interestingly, the overall estimated proportion of proviral sequences with *cis*-acting defects was found to be highest in PD-1^+^ cells compared to DN, DP and CTLA-4^+^ cells within this data (p<0.00001 for all; **Figure 2B**). However, this appeared to be driven by a single participant (PRA005). When this participant was removed, the DN to PD-1^+^ comparison was no longer significant, but the difference between PD-1^+^ and CTLA-4^+^, and PD-1^+^ and DP remained significant (p=0.0002 and p=0.026, respectively). PRA005 was also the only participant for which these types of sequences were identified in the CTLA-4^+^ cell subset (**Figure 2B**). We did not find evidence for differences in the overall proportion of proviruses with *cis*-acting defects between DN, CTLA-4^+^ and DP cells within this data (p>0.05 for all; **Table 1**). Altogether, these results suggest that proviruses with defects in the *cis*-acting region accumulate in PD-1^+^ cells compared to cells expressing CTLA-4. As all regions of the HIV-1 genome are intact except for the *cis*-acting region in these proviruses, this also indicates that PD-1^+^ cells containing these HIV-1 genomes also have a greater potential to express HIV-1 proteins compared to cell subsets that express CTLA-4.

Lastly, we investigated whether there is an enrichment for proviruses with an intact *p24* ORF in cells expressing PD-1, assuming that defective proviruses with an intact *p24* ORF are able to express p24 protein (34, 46, 47, 50). We therefore quantified the infection frequency of intact *p24* per 10^6^ cells within the four CD4^+^ T-cell subsets. We found that the infection frequency of proviruses with an intact *p24* ORF was highly variable between participants, with strong evidence that the observed differences in infection frequency between each of the cell subsets was highly dependent on participant-specific variation (**Table 1**). Consistent with previous work (27), however, we found that PD-1^+^ cells had the highest overall estimated infection frequency per 10^6^ cells of intact *p24* gene, followed by DN cells, DP cells and CTLA-4^+^ cells (**Figure 2C**). In general, we found strong evidence for PD-1^+^ cells having a higher intact *p24* infection frequency compared to DN cells, CTLA-4^+^ cells and DP cells within this data (all p<0.00001; **Figure 2C**). However, we also found strong evidence that these differences varied across participants (participant effect modification; p<0.00001 for PD-1^+^ vs DN and PD-1+ vs CTLA-4^+^, p=0.0005 for PD-1^+^ vs DP; **Table 1**). Moreover, we found evidence for a difference in the intact *p24* infection frequency between DN and DP cells (participant effect modification p=0.0003), CTLA-4^+^ cells and DP cells (participant effect modification p=0.05) and DN and CTLA-4^+^ cells (participant effect modification p=0.06), though these differences were variable across different participants (**Table 1**). Overall, this suggests that PD-1^+^ cells have high levels of proviruses with intact *p24*, but participant-specific differences play an important role in the accumulation of these proviruses in memory CD4^+^ T-cells.

To investigate whether differences in the infection frequency of proviruses with an intact *p24* ORF were influenced by similar factors to those that affect enrichment of all HIV-1 proviruses, we first calculated the infection frequency per 10^6^ cells of all HIV-1 proviruses (intact and defective; **Supplementary Figure 2**). We then investigated whether there was a correlation between total infection frequency and infection frequency of an intact *p24* gene (**Supplementary Figure 3**). For DN, PD-1^+^ and DP cells, we found a strong positive association between the total infection frequency and infection frequency of proviruses with intact *p24* (DN: r=0.78, 95% CI: 0.45, 0.92, p=0.0006; PD-1: r=0.96, 95% CI: 0.89, 0.99, p<0.0001; DP: r=0.78, 95% CI: 0.43, 0.92, p=0.001; **Supplementary Figure 3A, B, D**). In contrast, we observed that for CTLA-4^+^ cells there was no correlation between the total HIV-1 infection frequency and the infection frequency of proviruses with an intact *p24* gene (r=0.44, 95% CI: -0.11, 0.79, p=0.1; **Supplementary Figure 3C**). This indicates that there is a strong association between levels of total HIV-1 provirus and the ability of a HIV-1-infected cell to express p24, particularly in PD-1^+^ and CTLA-4^-^ cells.

Finally, to determine whether proviruses with an intact *p24* gene accumulate specifically in cells expressing PD-1, we calculated the proportion of sequences with an intact *p24* gene in each subset for each participant. Overall, we found evidence for PD-1^+^ cells having a higher proportion of sequences with an intact *p24* gene compared to CTLA-4^+^ cells (p=0.0001) and DP cells (p=0.003) within this data (**Figure 2D**), though, importantly, this difference varied across participants for PD-1^+^ vs CTLA-4^+^ (participant effect modification p=0.002; **Table 1**). This indicates that cells expressing CTLA-4 contain fewer proviruses with intact *p24*. We also found evidence for a difference in the proportion of proviruses with intact *p24* between DN cells and PD-1^+^ cells, but this varied across participants (participant effect modification p=0.01; **Table 1**). This finding indicates that in addition to having a higher infection frequency of proviruses with an intact *p24* gene, PD-1^+^ cells also have a higher proportion of proviruses with intact *p24* compared to cells expressing CTLA-4 (both single-positive and DP), which may explain why PD-1^+^ cells are able to express high levels of p24 protein upon *ex vivo* stimulation.

### Proviruses with genetically-intact *nef* are enriched in PD-1^+^ cells and less frequent in CTLA-4^+^ cells

**Figure 3:**
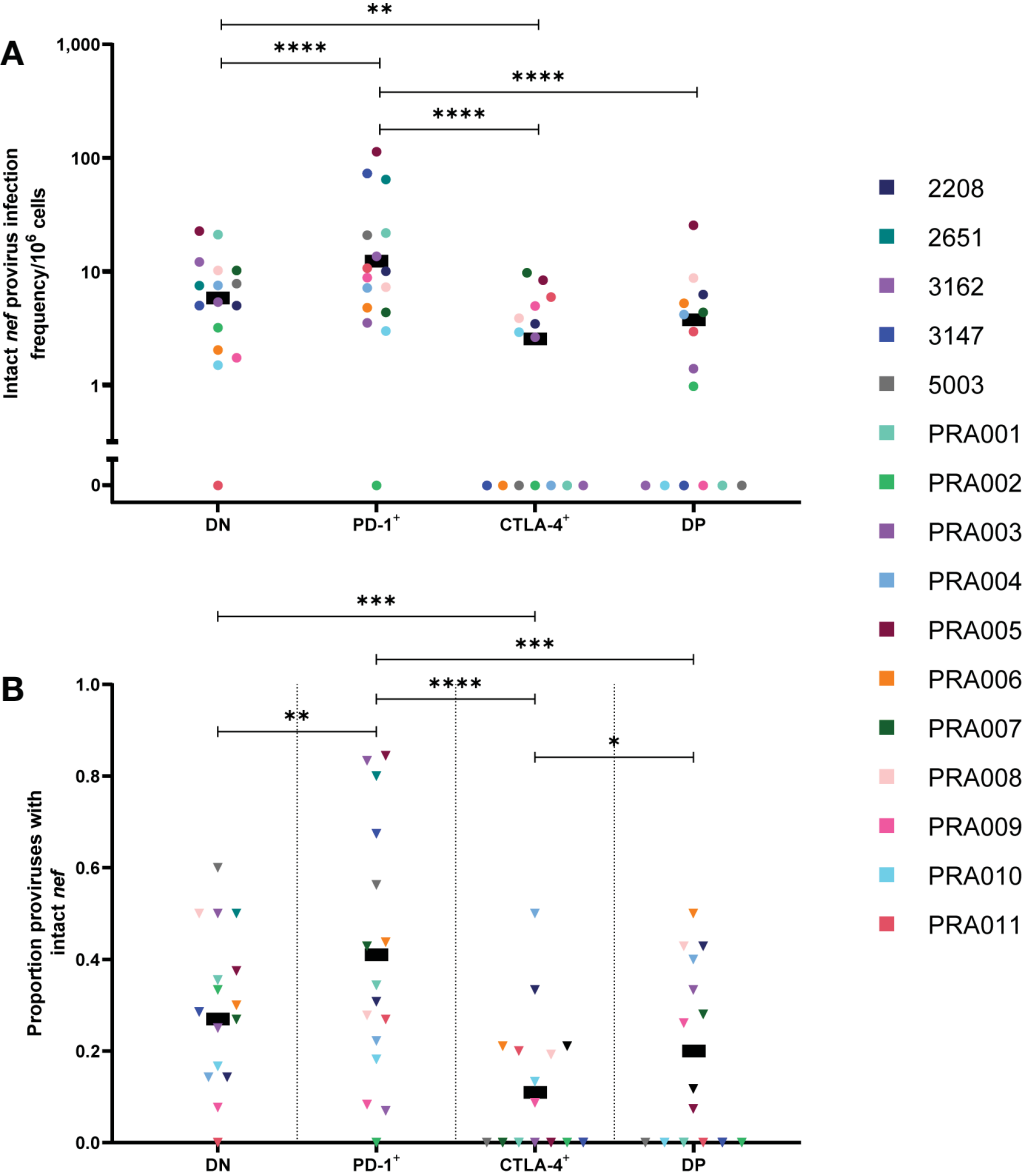
HIV-1 proviruses with an intact *nef* open reading frame in peripheral blood memory CD4^+^ T-cells expressing PD-1 and/or CTLA-4. The HIV-1 infection frequency per 10^6^ cells of proviruses with an intact *nef* ORF in DN, PD-1^+^, CTLA-4^+^ and DP cells **(A)**. The proportion of amplified proviruses that contained an intact *nef* ORF in DN, PD-1^+^, CTLA-4^+^ and DP cells **(B)**. Data is the estimated infection frequency per 10^6^ cells or proportion for each subset. p-values represent evidence for an overall trend of a difference between subsets within these participants (mixed effects logistic regression), * p ≤ 0.05, ** p ≤ 0.01, *** p ≤ 0.001, **** p ≤ 0.0001.

The HIV-1 protein Nef has been shown to have important functions in immune escape by downregulating expression of surface MHC-I, thereby allowing an HIV-1-infected cell to escape detection by HIV-1-specific CD8^+^ T-cells (46, 48, 51–53). We therefore investigated whether cells expressing PD-1 and/or CTLA-4 are enriched for proviruses containing a genetically-intact *nef* ORF. When comparing the infection frequency of proviruses with an intact *nef* ORF between the four cell subsets, we did find strong evidence that observed differences between these cell subsets were highly dependent on participant-specific variation in the magnitude and direction of the difference (**Table 1**). Overall, however, we observed that CTLA-4^+^ cells and DP cells had the lowest estimated infection frequency per 10^6^ cells of intact *nef* compared to the other cell subsets (**Figure 3A**). We found strong evidence that PD-1^+^ cells had a higher infection frequency per 10^6^ cells of intact *nef* compared to CTLA-4^+^ cells, DP cells and DN cells within this data (all p<0.00001; **Figure 3A**), with evidence that these differences varied across participants (participant effect modification; PD-1^+^ vs CTLA-4^+^: p=0.0001, PD-1^+^ vs DP: p=0.0095, and PD-1^+^ vs DN: p<0.00001; **Table 1**). Similarly, we found evidence for DN cells having a higher infection frequency of intact *nef* compared to CTLA-4^+^ cells (p=0.002) within this data (**Figure 3A**), with evidence that this varied across participants (participant effect modification p=0.02; **Table 1**). Together, this indicates that cells expressing CTLA-4 have a lower infection frequency of HIV-1 genomes containing an intact *nef* compared to cell types that don’t express CTLA-4.

We next utilised the calculated infection frequency of all HIV-1 proviruses (**Supplementary Figure 2**) to investigate whether intact *nef* infection frequency is correlated with total infection frequency for the four cell subsets (**Supplementary Figure 3**). A significant positive correlation was found between the total infection frequency and intact *nef* infection frequency for DN cells (r=0.65, 95% CI: 0.21, 0.87; p=0.008) and PD-1^+^ cells (r=0.87, 95% CI: 0.64, 0.95; p=<0.0001) (**Supplementary Figure 3A, B**). In contrast, no significant correlation was observed between total infection frequency and intact *nef* infection frequency for CTLA-4^+^ cells (r=0.32, 95% CI: -0.25, 0.72; p=0.25) and DP cells (r=0.23, 95% CI: -0.33, 0.67; p=0.41) (**Supplementary Figure 3C, D**).

To investigate whether proviruses with an intact *nef* ORF accumulate specifically in cells that don’t express CTLA-4, we compared the proportion of proviruses with intact *nef* between the four cell subsets. Complementing what was observed for the infection frequency of intact *nef*, we found strong evidence for DN cells having an overall higher proportion of proviruses with intact *nef* compared to CTLA-4^+^ cells within this data (p=0.0006; **Figure 3B**), with evidence that this varied across participants (participant effect modification p=0.006; **Table 1**). We also found evidence that PD-1^+^ cells have an overall higher proportion of proviruses with intact *nef* compared to CTLA-4^+^ cells (p<0.00001), DP cells (p=0.0001) and DN cells (p=0.002) within this data (**Figure 3B**), with evidence that this varied across participants for PD-1^+^ vs CTLA-4^+^ (participant effect modification p=0.004) and for PD-1^+^ vs DN (participant effect modification p=0.007) (**Table 1**). Interestingly, we also observed weak evidence for DP cells having a higher proportion of proviruses with intact *nef* compared to CTLA-4^+^ cells within this data (p=0.03; **Figure 3B**). We found no evidence for a difference in the proportion of proviruses with intact *nef* in DN cells compared to DP cells (p>0.05; **Table 1**).

### Proviruses with genetically-intact *tat* + *rev* + RRE are enriched in PD-1^+^ cells and less frequent in CTLA-4^+^ cells

**Figure 4:**
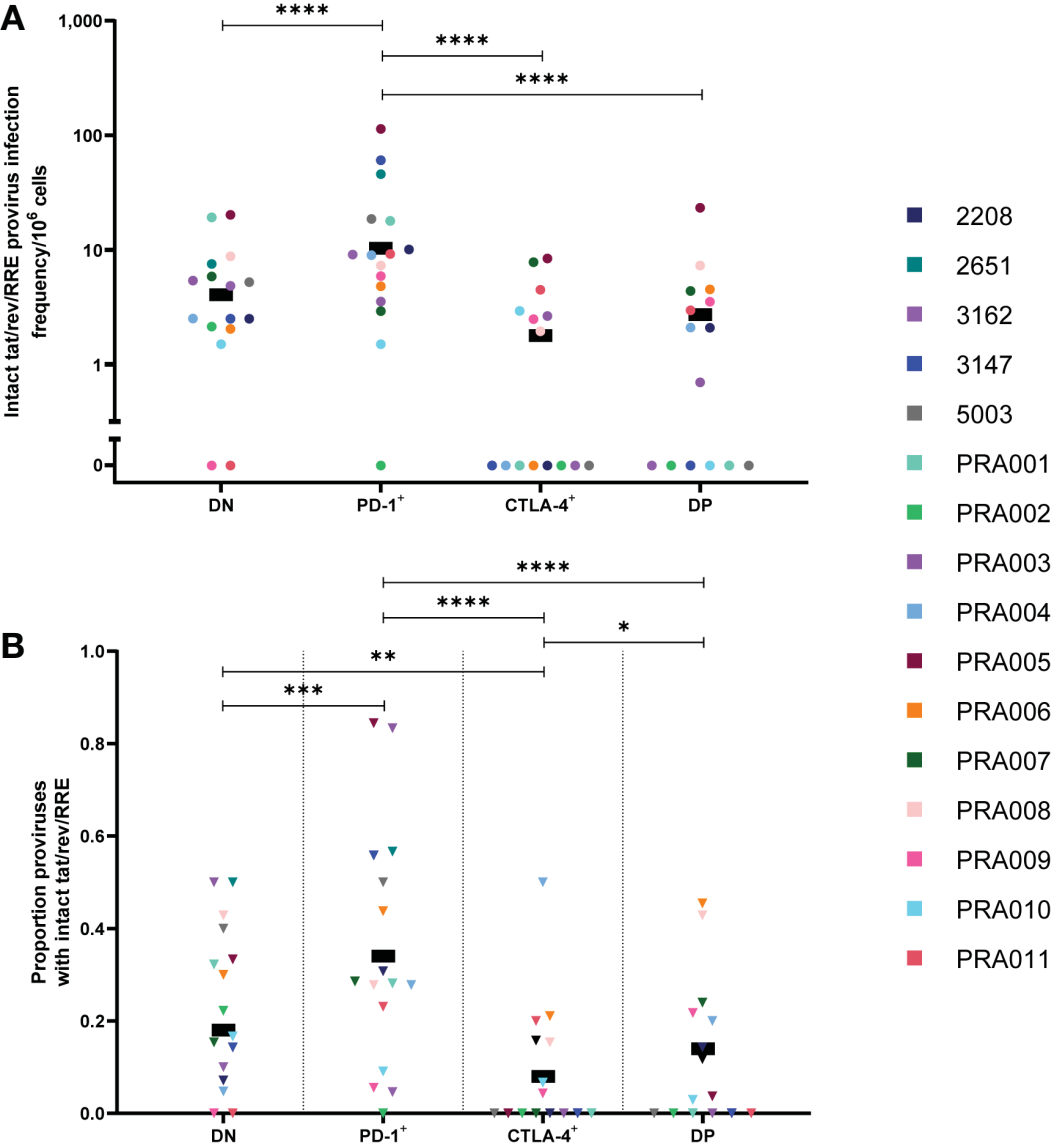
HIV-1 proviruses with an intact tat/rev/RRE^+^ open reading frame in peripheral blood memory CD4^+^ T-cells expressing PD-1 and/or CTLA-4. The HIV-1 infection frequency per 10^6^ cells of proviruses with an intact tat/rev/RRE^+^ ORF in DN, PD-1^+^, CTLA-4^+^ and DP cells **(A)**. The proportion of amplified proviruses that contained an intact tat/rev/RRE^+^ ORF in DN, PD-1^+^, CTLA-4^+^ and DP cells **(B)**. Data is the estimated infection frequency per 10^6^ cells or proportion for each subset. p-values represent evidence for an overall trend of a difference between subsets within these participants, * p ≤ 0.05, ** p ≤ 0.01, *** p ≤ 0.001, **** p ≤ 0.0001.

The HIV-1 viral proteins Tat and Rev are essential in the production of partially-spliced and unspliced RNA transcripts during HIV-1 proviral expression, and the Rev response element (RRE) is essential in ensuring that partially-spliced and unspliced RNA transcripts are able to leave the nucleus of an infected cell for translation (54). Previous studies of anti-PD-1 and anti-PD-1/anti-CTLA-4 combination immune blockade in HIV-1-infected individuals and SIV-infected non-human primates have indicated an increase in cell-associated unspliced RNA expression in response to this treatment (16, 19, 22–24). We therefore investigated whether these results may be due to increased levels of proviruses with intact tat/rev/RRE ORFs (referred to as tat/rev/RRE^+^) in cells expressing PD-1 and/or CTLA-4. Though we did find significant evidence for a strong contribution of participant-specific variation to any enrichment of proviruses with intact tat/rev/RRE^+^ ORFs within specific cell subsets (**Table 1**), overall we observed that the estimated infection frequency of proviruses with intact tat/rev/RRE was highest in PD-1^+^ cells, followed by DN cells, DP cells and CTLA-4^+^ cells (**Figure 4A**). We found strong evidence that PD-1^+^ cells had the highest infection frequency of tat/rev/RRE^+^ proviruses compared to DN cells, CTLA-4^+^ cells and DP cells within this data (p<0.00001 for all; **Figure 4A**), however with strong evidence that these differences did vary across participants for PD-1^+^ vs DN (participant effect modification p=0.0001) and PD-1^+^ vs CTLA-4^+^ (participant effect modification p=0.0005) (**Table 1**). We found no evidence for a difference between DN and DP cells, or CTLA-4^+^ cells and DP cells within this data (p>0.05 for both; **Table 1**). Altogether, this indicates that PD-1^+^ cells are enriched for intact tat/rev/RRE compared to cells that don’t express PD-1 or those that express CTLA-4, which may lead to increased transcription initiation, reflected by higher levels of cell-associated unspliced RNA.

To further investigate the role of PD-1 as a marker for tat/rev/RRE^+^ proviruses, we utilised the calculated overall proviral infection frequency within each of the cell subsets (**Supplementary Figure 2**) to look for correlations between the total infection frequency and the infection frequency of tat/rev/RRE^+^ proviruses in the four cell subsets (**Supplementary Figure 3**). We found a significant positive correlation between the total infection frequency and the infection frequency of tat/rev/RRE^+^ proviruses in PD-1^+^ cells (r=0.78, 95% CI: 0.45, 0.92, p=0.0006; **Supplementary Figure 3B**) and DP cells (r=0.56, 95% CI: 0.06, 0.84, p=0.03; **Supplementary Figure 3D**). The correlation between total infection frequency and the infection frequency of tat/rev/RRE^+^ proviruses in DN cells approached significance (r=0.5, 95% CI: -0.01, 0.8, p=0.05; **Supplementary Figure 3A**), while no significant correlation was observed between total infection frequency and the infection frequency of tat/rev/RRE^+^ proviruses in CTLA-4^+^ cells (r=0.25, 95% CI: -0.32, 0.68, p=0.37; **Supplementary Figure 3C**). This indicates that PD-1 may be a marker for HIV-1-infected cells with tat/rev/RRE^+^ proviral genomes, while CTLA-4 may be a marker for reduced frequency of tat/rev/RRE^+^ proviruses during suppressive ART.

To determine whether proviruses that are tat/rev/RRE^+^ specifically accumulate in cells expressing PD-1, we compared the proportion of proviruses that are tat/rev/RRE^+^ between the four cell subsets. We again observed evidence that differences in the proportion of tat/rev/RRE^+^ proviruses between the four cell subsets were participant-specific (**Table 1**). Overall, however, we did find evidence that PD-1^+^ cells had the highest proportion of proviruses that are tat/rev/RRE^+^ compared to DN cells (p=0.0001), CTLA-4^+^ cells (p<0.00001) and DP cells (p<0.00001) within this data (**Figure 4B**), with evidence that these differences varied across participants for PD-1^+^ vs DN (participant effect modification p=0.02) and PD-1^+^ vs CTLA-4^+^ (participant effect modification p=0.04) (**Table 1**). We also found evidence that DN cells had a higher proportion of proviruses that are tat/rev/RRE^+^ compared to CTLA-4^+^ cells within this data (p=0.006; **Figure 4B**), with evidence that this varied across participants (participant effect modification p=0.03; **Table 1**). Interestingly, we also observed evidence that DP cells have a higher proportion of proviruses with intact tat/rev/RRE compared to CTLA-4^+^ cells within this data (p=0.03; **Figure 4B**). We found no evidence of a difference in the proportion of proviruses with intact tat/rev/RRE between DN and DP within this data (p=0.46; **Figure 4B**). This further indicates that PD-1 expression may be a marker for proviruses with intact tat/rev/RRE ORFs.

### Peripheral blood PD-1^+^ cells have a high proportion of genetically-identical sequences

**Figure 5:**
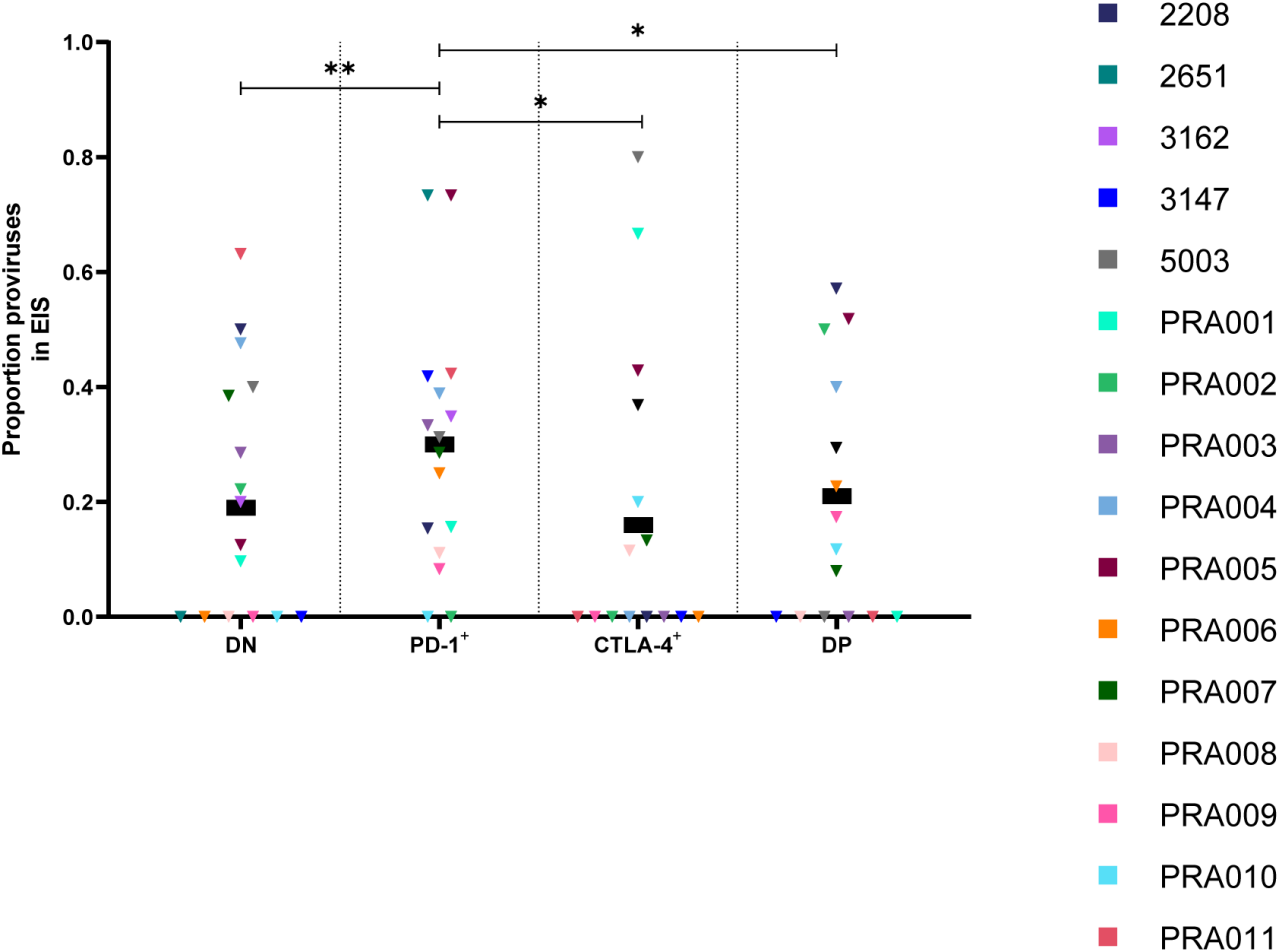
Proportion of HIV-1 proviruses found in an expansion of identical sequences (EIS) in peripheral blood memory CD4^+^ T-cells expressing PD-1 and/or CTLA-4. The proportion of amplified HIV-1 proviruses that were part of an EIS in DN, PD-1^+^, CTLA-4^+^ and DP cells. Data is the estimated proportion for each subset. P-values represent evidence for an overall trend of a difference between subsets within these participants (mixed effects logistic regression), * p ≤ 0.05, ** p ≤ 0.01.

The role of cellular proliferation in the maintenance of the HIV-1 reservoir during suppressive ART is well-known (10, 25, 37, 48, 55–64). Groups of two or more sequences within a cell subset that are 100% identical to one another are defined as an expansion of identical sequences (EIS) (37). When we investigated the proportion of sequences within each cell subset that were part of an EIS in the peripheral blood, we found evidence that all differences between cell subsets were affected by participant-specific variation (**Table 1**). Overall within these participants, however, we observed that PD-1^+^ cells have the highest estimated proportion of sequences that are part of an EIS, followed by DP cells, DN cells and CTLA-4^+^ cells (**Figure 5**). We found evidence that PD-1^+^ cells had a higher proportion of sequences part of an EIS compared to CTLA-4^+^ cells (p=0.02) and DN cells (p=0.001) within this data (**Figure 5**), with strong evidence that these differences varied across participants (participant effect modification p<0.00001 for both; **Table 1**). We also found weak evidence that PD-1^+^ cells had a higher proportion of sequences part of an EIS compared to DP cells (p=0.05; **Figure 5**) within this data, with evidence that this difference varied across participants (participant effect modification p=0.01; **Table 1**). We found evidence for a difference in the proportion of sequences that were part of an EIS between DN and CTLA-4^+^ cells (participant effect modification p=0.0001), DN and DP cells (participant effect modification p=0.03), and CTLA-4^+^ cells and DP cells (participant effect modification p=0.008), though these differences were highly dependent on the participant (**Table 1**). Altogether, this suggests that PD-1^+^ cells harbour a high level of identical proviral sequences, which is likely to contribute to the persistence of higher levels of HIV-1 proviruses with certain intact ORFs.

### Lymph node CTLA-4^+^ cells have low levels of HIV-1 provirus

**Figure 6:**
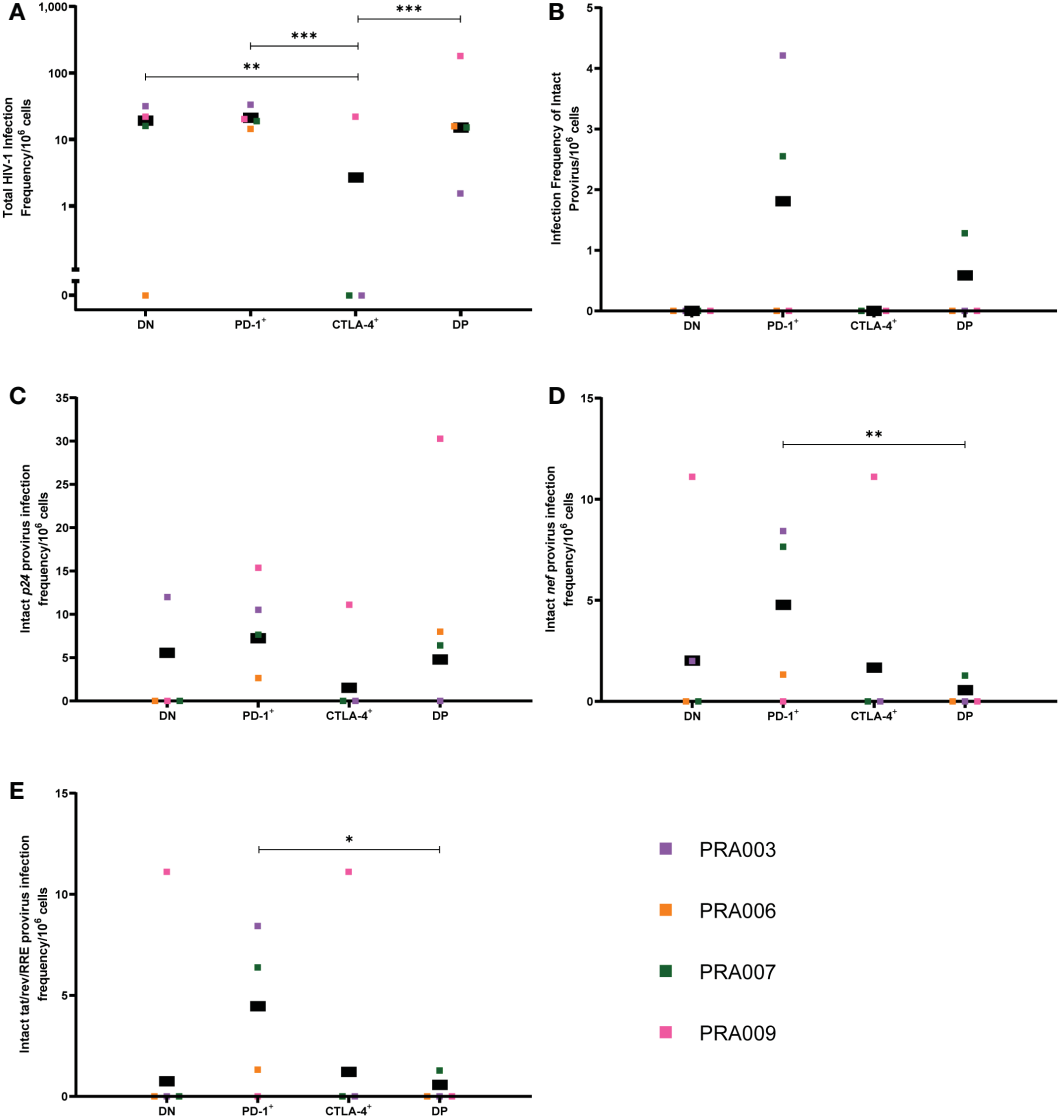
Frequency of HIV-1 proviruses in lymph node memory CD4^+^ T-cells expressing PD-1 and/or CTLA-4. The HIV-1 infection frequency per 10^6^ cells of total HIV-1 proviruses **(A)**, genetically-intact proviruses **(B)**, proviruses with an intact *p24* ORF **(C)**, proviruses with an intact *nef* ORF **(D)** and proviruses with an intact tat/rev/RRE ORF **(E)** in DN, PD-1^+^, CTLA-4^+^ and DP cells from the lymph node. Data is the estimated infection frequency per 10^6^ cells for each subset. p-values represent evidence for an overall trend of a difference between subsets within these participants (mixed effects logistic regression), * p ≤ 0.05, ** p ≤ 0.01, *** p ≤ 0.001.

In addition to the peripheral blood samples, we also had access to lymph node biopsies for four participants. Memory CD4^+^ T-cells from the lymph node were sorted into the same four cell subsets (DN, PD-1^+^, CTLA-4^+^ and DP cells) as investigated in the peripheral blood, and FLIPS was utilised to genetically-characterise the HIV-1 provirus within these cell subsets. We note that the low number of cells available for sequencing from these cell subsets in the lymph node compared to what was available from the peripheral blood (**Table 2**) has restricted the depth of our analyses. When we compared the total HIV-1 infection frequency per 10^6^ cells within the lymph node, similar to the peripheral blood, we observed that many differences in infection frequencies between the four cell subsets were affected by substantial participant-specific variation (**Table 1**). Overall, however, we found that the estimated total infection frequency was higher in DN, PD-1^+^ and DP cells compared to CTLA-4^+^ cells (**Figure 6A**). We found evidence that, overall, CTLA-4^+^ cells had a lower total infection frequency of HIV-1 in the lymph node compared to DN cells (p=0.006), PD-1^+^ cells (p=0.001) and DP cells (p=0.0002) within this data (**Figure 6A**), with evidence that these differences varied across participants for PD-1^+^ vs CTLA-4^+^ (participant effect modification p=0.03) and DN vs CTLA-4^+^ (participant effect modification p=0.05) (**Table 1**). We also found evidence for a difference in the total infection frequency between DN and DP cells, and PD-1^+^ and DP cells, but these differences were highly variable across participants (participant effect modification p<0.00001 for both; **Table 1**). We found no evidence for a difference in the total infection frequency between DN cells and PD-1^+^ cells in the lymph node within this data (p=0.61; **Table 1**). Altogether, this suggests that CTLA-4 expression in the lymph node is a marker for lower levels of HIV-1 provirus, with a potential role for co-expression with PD-1 leading to increased levels of HIV-1 provirus in some participants.

We were only able to identify genetically-intact proviruses within the lymph node for two out of four participants, with these intact proviruses found in the PD-1^+^ and DP cell subsets (**Figure 6B**). We therefore found no statistically significant difference in the infection frequency of genetically-intact provirus, or proportion of proviruses that were genetically-intact, between cell subsets expressing PD-1 and/or CTLA-4 in the lymph node (**Table 1**). Similarly, we were unable to identify any overall statistically significant differences in the infection frequency of proviruses with an intact *p24* ORF between the four cell subsets within this data, though we observed that DN, PD-1^+^ and DP cells had a higher estimated infection frequency of provirus with an intact *p24* ORF compared to CTLA-4^+^ cells (**Figure 6C**). We did find evidence for a difference in the intact *p24* infection frequency between DN and DP cells (participant effect modification p=0.001) and PD-1^+^ and DP cells (participant effect modification p=0.02), but these differences were highly dependent on the participant (**Table 1**).

When considering the infection frequency of proviruses with an intact *nef* gene, we found evidence that, overall, PD-1^+^ cells have a higher intact *nef* infection frequency compared to DP cells within this data (p=0.006; **Figure 6D**). We did not observe any statistically significant difference in the intact *nef* infection frequency between any other cell subset. Similarly, for the infection frequency of proviruses with intact tat/rev/RRE, we only observed a significantly higher infection frequency of tat/rev/RRE^+^ proviruses for PD-1^+^ cells compared to DP cells within this data (p=0.01; **Figure 6E**).

In summary, our findings suggest that in the lymph node, CTLA-4^+^ cells have a lower HIV-1 infection frequency compared to CTLA-4^-^ cells, and particularly PD-1^+^ cells. These PD-1^+^ cells also have higher levels of proviruses with intact *p24* and intact *nef* ORFs, which may be indicative of the presence of genetically-intact genomes in PD-1^+^ cells.

### Adjustment for multiple comparisons

The 312 comparisons presented in **Table 1** and **Supplementary Table 2** will have higher family wise type 1 error rates than that suggested by any single p-value. To investigate this, we calculated worst-case false discovery rates at different p-value value thresholds to help understand the possibility of spurious results. These are “worst-case” as all comparisons in **Supplementary Table 2** are treated as independent comparisons, when many of these are in fact correlated (e.g. intact infection frequency and intact *p24*, *nef*, *tat*, or *rev* ORFs). We find that for a p-value thresholds of 0.05, 0.02, 0.01 and 0.001, the worst-case false discovery rate (expected proportion of potentially spurious results) is 0.13, 0.066, 0.039, and 0.005, respectively. These worst-case false discovery rates are not considered unacceptably high. However, we stress that due to the exploratory nature of our study and the possibility of spurious results, our findings presented here have identified areas that require further investigation in future studies in order to confirm the distribution of HIV-1 proviruses in CD4^+^ T cells expressing PD-1 and/or CTLA-4 in HIV-1-infected individuals on long-term ART.

## Discussion

In this exploratory study, we have used the FLIPS assay (10) to sequence near-full-length proviral genomes from CD4^+^ T-cells that have been sorted based on the expression of PD-1 and/or CTLA-4. We found that in the peripheral blood, PD-1^+^ cells harbored high levels of proviruses with intact ORFs for specific genomic regions such as *p24*, *nef* and *tat*/*rev.* However, many of the differences in infection frequencies of proviral genomes between these cell subsets were participant-specific, both in the extent and direction of the difference. We also found that in the peripheral blood, CTLA-4 is a marker for reduced levels of genetically-intact HIV-1 provirus, as well as those with specific ORFs intact. In addition, we found that lymph node CTLA-4^+^ cells have a lower HIV-1 infection frequency compared to the other cell subsets. These findings have highlighted that further work should investigate PD-1, rather than CTLA-4, as a marker for higher levels of HIV-1 proviruses, including genetically-intact proviruses, in HIV-1-infected individuals on long-term ART.

Our studies of the distribution of HIV-1 proviruses with particular intact ORFs within cell subsets sorted based on PD-1 and CTLA-4 expression have revealed substantial participant-specific variation in the infection frequencies of these proviruses, and in the cell subsets which are enriched for certain HIV-1 genomes. In particular, we found considerable participant-specific variation in the enrichment of certain HIV-1 genomes when comparing PD-1^+^ cells to each of the other cell subsets. This variation revealed that some participants had significantly more HIV-1 genomes within the PD-1^+^ cell subsets than the DN, CTLA-4^+^ and DP subsets, while other participants have little or no difference between these subsets. This indicates that PD-1 expression may only be a marker for increased HIV-1 infection in some HIV-1-infected participants. This variation may be affected by factors such as the length of time the participants have been infected with HIV-1, the timing of their ART initiation, or the length of time they have been on ART (62, 65–68). However, much of this information was not available for all of these participants, though the length of time the participants have been virally suppressed was variable between participants (**Supplementary Table 1**). Additional clinical parameters, such as those listed in **Supplementary Table 1**, may also contribute to the strong participant-specific effect observed in this study. Due to the low number of genetically-intact proviruses isolated in this study, we did not investigate whether clinical parameters correlated with the size of the genetically-intact HIV-1 reservoir in these participants. However, Rasmussen et al. (2022) did perform a comparison between clinical parameters and total HIV-1 DNA content in these participants (31). Significant correlations were only observed between total HIV-1 DNA content and CD8^+^ T cell count and percentage, and this was observed in DN and CTLA-4^+^ cells only in the peripheral blood. An additional possible contributing factor to the participant-specific effect modification in our results may be the large variation in available cell numbers and sequenced genomes between cell subsets and participants (see **Table 2**). Previous studies have also indicated that there is substantial variability between participants in identifying the subsets that contribute to HIV-1 persistence (10, 25, 37, 55, 60, 69). Furthermore, multiple studies have indicated a strong positive correlation between levels of PD-1 expression on CD4^+^ T-cells and levels of HIV-1 DNA in ART-suppressed participants (15, 26, 70). Although these studies did not investigate this correlation specifically in CD4^+^ T-cells that express PD-1, these results do indicate that participant-specific differences relating to PD-1 expression levels influence the level of persistent HIV-1 present in the peripheral blood. CD4^+^ T-cells that would express PD-1 are likely to include EM cells (25, 26, 71) and CXCR5^+^PD-1^+^ circulating Tfh (cTfh) cells, which have been shown to be enriched for total and potentially replication-competent HIV-1 DNA (10, 27, 28, 72). It is therefore likely that the peripheral blood PD-1^+^ and DP compartments within our study include some crossover with cells expressing additional markers such as CXCR5 and markers for EM cells, and this crossover may partially explain the large role of participant-specific differences in the enrichment of HIV-1 genomes within PD-1^+^ cells. Much of the previous work identifying the enrichment of HIV-1 DNA within PD-1^+^ cells have observed this in the context of additional cellular markers, such as on CM, TM or EM cells (15, 25, 26). For example, Fromentin et al. (2019) demonstrated enrichment of integrated HIV-1 DNA in PD-1^+^ cells compared to PD-1^-^ cells in CM and TM cells, while no enrichment was seen in EM cells (15). Altogether, this indicates that though our work has shown some enrichment of genetically-intact HIV-1 provirus in PD-1^+^ cells, it is likely that additional markers are needed to consistently identify HIV-1-infected cells within ART-suppressed participants.

Our results indicate that in the peripheral blood, CTLA-4 expression may be a marker for lower levels of genetically-intact HIV-1 provirus and proviruses with certain genetic characteristics. We found that both DN and PD-1^+^ cells, the subsets that do not express CTLA-4, had a higher infection frequency of genetically-intact HIV-1 provirus compared to CTLA-4^+^ and DP cells (that express CTLA-4). This is in contrast to a previous study by McGary et al. (2017) that indicated enrichment of SIV DNA in peripheral blood CTLA-4^+^ cells compared to DN (PD-1^-^CTLA-4^-^) and DP (PD-1^+^CTLA-4^+^) cells during ART treatment (30). However, our finding that DP cells have lower levels of genetically-intact HIV-1 compared to DN cells supports the findings of Rasmussen et al. (2022), who observed that DP cells were more resistant to the induction of multiply-spliced HIV-1 RNA expression compared to DN cells despite considerable total HIV-1 DNA levels (31). The low level of genetically-intact provirus in DP cells provides an additional possible explanation for this finding as defective proviruses will be less likely to express multiply-spliced HIV-1 RNA (31). Our data has suggested that defective proviruses accumulate in CTLA-4^+^ cells compared to CTLA-4^-^ and PD-1^+^ cells, with these CTLA-4^+^ cells having the highest proportion of proviruses with large internal deletions. In addition, we found cells that express CTLA-4 have lower levels of proviruses with intact *p24*, *nef* and *tat/rev* ORFs. These defective proviruses are able to persist due to the lack of HIV-1 protein expression, allowing them to avoid being targeted by HIV-1-specific CD8^+^ T-cells (45, 46, 48, 49, 57, 67). We do acknowledge that we had low numbers of CTLA-4^+^ cells in some participants, which is reflective of the low proportion of CTLA-4^+^ cells within the peripheral blood and lymph node memory CD4^+^ T-cell compartment in these participants as measured by Rasmussen et al. (2022) (31). This may have skewed our results and limited our ability to identify HIV-1 provirus in CTLA-4^+^ and DP cells, as we have included these cells based on cell surface expression of CTLA-4. The known cycling of CTLA-4 expression between the cell surface and endosomal vesicles makes the capture of transient cell surface CTLA-4 expression difficult (73, 74). The study by McGary et al. (2017) did utilize PMA/ionomycin activation of CD4 T cells prior to cell sorting to increase the detection of surface CTLA-4 expression (30). Our study addressed this possible limitation by staining cells for CTLA-4 expression for an extended period of time prior to other cell surface markers, which was shown to detect similar surface CTLA-4 expression levels compared to the activation of cells at room temperature prior to sorting, while limiting the potential for altering the phenotype, and particularly the activation status, of the cells or reactivating latent HIV-1 within these cells (31). We also acknowledge that we have not compared the total CTLA-4^+^ CD4^+^ T-cell subset with the total CTLA-4^-^ compartment, which would further elucidate whether CTLA-4 expression may be a marker for genetically-defective HIV-1 proviruses. Altogether, our findings indicate that CTLA-4^+^ cells have reduced levels of genetically-intact HIV-1 provirus in the peripheral blood, as well as proviruses with specific intact HIV-1 ORFs.

Previous work by Pardons et al. (2019) found that during suppressive ART, PD-1^+^ cells are enriched for proviruses that can express p24 protein in response to stimulation (27). Additionally, Baxter et al. (2016) found that during untreated HIV-1 infection, T-cells expressing HIV-1 RNA and Gag protein were enriched particularly in T-cells expressing PD-1 and Tfh cell markers (PD-1^+^CXCR5^+^), with additional enrichment in cells co-expressing PD-1 with other immune checkpoint markers (TIGIT^+^ and CTLA-4^+^) (28). We, however, did not see an overall enrichment of genetically-intact provirus in PD-1^+^ cells compared to DN cells, which don’t express PD-1. We did find that PD-1^+^ cells had the highest level of provirus with an intact *p24* ORF, which may include both genetically-intact proviruses and proviruses with genetic defects outside of the *p24* ORF. Several recent studies have shown that defective proviruses are able to express p24 protein, as well as other HIV proteins (34, 46, 47, 50). However, we acknowledge that we have not compared the total PD-1^+^ cell subset to total PD-1^-^ cells, which may be more reflective of the cells included in these previous studies. Altogether, these results provide further evidence that the expression of HIV-1 p24 protein does not necessarily indicate the presence of replication-competent provirus within a cell.

The expression of the HIV-1 Nef protein contributes to HIV-1 persistence within infected cells by downregulating the expression of surface MHC-I, thereby allowing an HIV-1-infected cell to escape detection by HIV-1-specific CD8^+^ T-cells (46, 48, 51–53). We observed that CTLA-4 expression may be a marker for reduced levels of proviruses with an intact *nef* ORF, with evidence for DN and PD-1^+^ cells having an overall higher infection frequency of intact *nef* compared to CTLA-4^+^ and DP cells. This increased level of proviruses with intact *nef* ORFs may allow the preferential persistence of HIV-1 proviruses in PD-1^+^ cells during ART. It has been shown *in vitro* that the HIV-1 Nef protein can downregulate CTLA-4 expression by 57-77% during productive infection (29, 75). Conversely, Nef can induce the expression of PD-1 on the surface of CD4^+^ T-cells *in vitro* (76). Recent studies have shown that the HIV-1 Nef protein is expressed during ART and can be produced from defective proviruses (46, 47, 77, 78). Therefore, the presence of the HIV-1 Nef protein during ART may affect PD-1 and CTLA-4 expression on infected cells, in addition to the persistence of these infected cells. In agreement with this possible effect of Nef, we found that the infection frequency of intact *nef* was highly correlated with the HIV-1 infection frequency of DN and PD-1^+^ cells, but not significantly correlated with the HIV-1 infection frequency of CTLA-4^+^ and DP cells. This indicates that there is only a strong association between levels of HIV-1 provirus and intact *nef* for cells that do not express CTLA-4, suggesting a possible negative effect of Nef on CTLA-4 expression *in vivo*. In summary, our results indicate that the expression of Nef protein may contribute to the persistence of HIV-1 genomes in PD-1^+^ cells.

In addition to this enrichment for intact *nef* ORFs in PD-1^+^ cells compared to cell subsets expressing CTLA-4, we also found evidence that PD-1 may be a marker for HIV-1-infected cells with tat/rev/RRE^+^ proviruses. The initiation of unspliced HIV-1 RNA transcription is increased in the presence of Tat and Rev protein expression (54), indicating that substantial amounts of unspliced cell-associated HIV-1 RNA would theoretically only be expressed from proviruses with these ORFs intact. Previous studies of anti-PD-1 and anti-PD-1/anti-CTLA-4 combination immune blockade in HIV-1-infected individuals and SIV-infected non-human primates showed increases in cell-associated unspliced RNA expression (16, 19, 22–24). Our observed results that PD-1^+^ cells are enriched for proviruses with intact tat/rev/RRE^+^ ORFs would indicate that during anti-PD-1 immune blockade, these cells could produce unspliced HIV-1 RNA, in agreement with these immune blockade treatment studies.

The role of cellular proliferation in the persistence of the HIV-1 reservoir is well-known (10, 25, 37, 48, 55–64). In the current study, we observed that PD-1^+^ cells have a higher proportion of sequences that are part of an expansion of identical sequences (EIS) compared to DN cells, DP cells and CTLA-4^+^ cells, suggesting that PD-1^+^ cells have the highest level of cellular proliferation compared to the other subsets explored in this study. This is consistent with the fact that PD-1 is considered a cellular activation marker, in addition to its role in immune exhaustion (79). Chomont et al. (2009) and Fromentin et al. (2016) both found that the level of PD-1 expression on CD4^+^ T-cells was highly correlated with the level of Ki67 expression, a marker of cellular proliferation, on CD4^+^ T-cells (25, 26), perhaps indicating that the level of PD-1 expression observed on memory CD4^+^ T-cells within these participants may influence the level of cellular proliferation, though we have not performed this measurement. We also observed that the level of cellular proliferation was highly participant-specific, indicating that different HIV-1-infected individuals have different levels of cellular proliferation contributing to their latent HIV-1 reservoir. We acknowledge that the high proportion of sequences that are part of an EIS in PD-1^+^ cells have likely contributed to the high levels of cells with intact *p24*, *nef* and tat/rev/RRE^+^ ORFs within the PD-1^+^ compartment discussed earlier. However, Duette et al. (2022) observed a high level of intact *nef* in EM cells, even after removing identical sequences from the analysis. This indicates that Nef plays a role in the persistence of HIV-1 proviruses in EM cells, which contain a large proportion of PD-1^+^ cells (25, 26, 71), even after the role of cellular proliferation has been excluded (46). Our results therefore reveal that high levels of cellular proliferation in many ART-suppressed participants may contribute to the persistence of HIV-1 proviruses in PD-1^+^ cells during suppressive ART.

In the lymph node, we found evidence that CTLA-4^+^ cells had a lower total HIV-1 infection frequency compared to DN cells, PD-1^+^ cells and DP cells, similar to our observations in the peripheral blood. However, these differences in total HIV-1 infection frequency between cells expressing PD-1 and/or CTLA-4 within the lymph node were often affected by participant-specific differences. Banga et al. (2016) showed that a mean of 65% of the lymph node PD-1^+^ CD4^+^ T-cell compartment within ART-suppressed HIV-1-infected individuals was made up of PD-1^+^CXCR5^+^ Tfh cells. These Tfh cells have been shown to be a reservoir for inducible replication-competent HIV-1 in untreated and ART-suppressed HIV-1-infected individuals, though there was a large amount of participant variability (8, 9). It is likely, therefore, that some of these participant-specific differences in the lymph node observed in our study may be due to differences in the proportion of lymph node CD4^+^ T-cells that are made up by PD-1^+^CXCR5^+^ Tfh cells. We note that due to the low number of genetically-intact proviruses identified within the lymph node, we were unable to identify statistically significant differences in the infection frequency or proportion of genetically-intact proviruses between the cell subsets. However, the genetically-intact proviruses we were able to identify in the lymph node were found within the PD-1^+^ and DP subsets. Based on the previous work of Banga et al. and Perreau et al., it is likely that these genetically-intact proviruses were found within lymph node Tfh cells (8, 9, 72). We also acknowledge that the availability of lymph node samples from only four participants does limit the generalizability of these results to the greater ART-suppressed HIV-1-infected population.

We acknowledge that there are several limitations to our study. Firstly, due to the transient nature of CTLA-4 cell surface expression (73), it is likely that the CTLA-4^+^ compartments within our study are an underestimate of the true proportion of cells that express CTLA-4. Similarly, we also acknowledge that despite the high purity of the sorted cell populations (see Methods) assessed in this study, there is a possibility that there is contamination of DN cells within either of the PD-1^+^ or CTLA-4^+^ gates. Secondly, the number of cells available from some participants, particularly in the lymph node samples and in the peripheral blood CTLA-4^+^ and DP compartments, did restrict the number of sequenced proviruses, as well as our ability to identify EIS within these cell subsets. Our statistical analysis aims to consider this limitation, though it is likely that the depth of our sampling has reduced the power of our results in some areas. Thirdly, we have performed a large number of statistical comparisons in this exploratory study, which raises the possibility of false-significant p-values. We estimated the worse-case false discovery rate of this, and did not find it to be unacceptably high. However, the results should still be treated as exploratory in nature whose value is to identify areas in need of future exploration. Fourth, the known low amplification efficiency of near-full-length HIV-1 genomes (80) will likely have impacted our ability to identify all genetically-intact, and indeed full-length, proviruses within the participant samples included in our study (see **Table 2**). However, this is unlikely to have impacted our ability to compare between individual cell subsets within individual participants. We also have not investigated the infectiousness of the identified genetically-intact proviruses, and we therefore cannot conclude that these are truly replication-competent. Moreover, the participants included in this study are all biologically male, and future studies will therefore be needed to determine how biological sex contributes to the differences seen between cells expressing PD-1 and/or CTLA-4, as has been seen in other studies of HIV-1 pathogenesis (81, 82). Finally, 15/16 participants included in this study are infected with HIV-1 subtype B, while 1 participant is infected with subtype CRF01_AE. Future work will need to focus on additional subtypes in characterizing the cell types harboring latent HIV-1 provirus.

In summary, our exploratory analysis has indicated that PD-1^+^ cells have the characteristics which contribute to HIV-1 persistence during ART, such as high proliferative capability, activation status, and genetically-intact regions encoding viral proteins, including Tat and Nef, whose expression allows for immune evasion. Conversely, we identified that CTLA-4 expression is a marker for HIV-1 provirus that is more likely to be defective, and contain low levels of these intact ORFs. We do stress, however, that future work is required to confirm these results. Importantly, we also identified that participant-specific variation contributed to most differences in the levels of particular HIV-1 proviruses found between memory CD4^+^ T-cells expressing PD-1 and/or CTLA-4. This indicates that the search for a single specific cell surface marker that identifies persistent HIV-1-infected cells is complicated by considerable variation between individual participants in the cell types that contribute to the HIV-1 reservoir. As a result, consideration of multiple additional cellular markers will likely be needed to consistently identify cells harboring latent, and potentially replication-competent, HIV-1 in the general population.

## Data availability statement

The datasets presented in this study can be found in online repositories. The names of the repository/repositories and accession number(s) can be found below: https://www.ncbi.nlm.nih.gov/genbank/, OP700895-OP701628.

## Ethics statement

The studies involving human participants were reviewed and approved by the institutional review board at the Western Sydney Local Health District, which includes the Westmead Institute for Medical Research, the Human Research Ethics Committees at The Alfred and Avenue Hospitals in Melbourne, the University of Melbourne Ethics Committee, and the Institutional Review Board at the University of California San Francisco. The patients/participants provided their written informed consent to participate in this study.

## Author contributions

Conceptualization, KF, TAR, SRL and SP; Methodology, KF, SRL and SP; Formal Analysis, KF, TES and ZB; Investigation, KF and ZB; Resources, TAR, RH, FH, SGD, SRL and SP; Data Curation, KF; Writing – Original Draft, KF; Writing – Review and Editing, TES, TAR, SRL and SP; Visualization, KF; Supervision, SP; Project Administration, TAR, AR, SRL and SP; Funding Acquisition, SGD, SRL and SP. All authors contributed to the article and approved the submitted version.
