# Supplementary figures and images for: Unequal distribution of genetically-intact HIV-1 proviruses in cells expressing the immune checkpoint markers PD-1 and/or CTLA-4
Source: Front Immunol. 2023 Jan 26;14:1064346. doi: 10.3389/fimmu.2023.1064346 (PMC9909745; doi:10.3389/fimmu.2023.1064346)

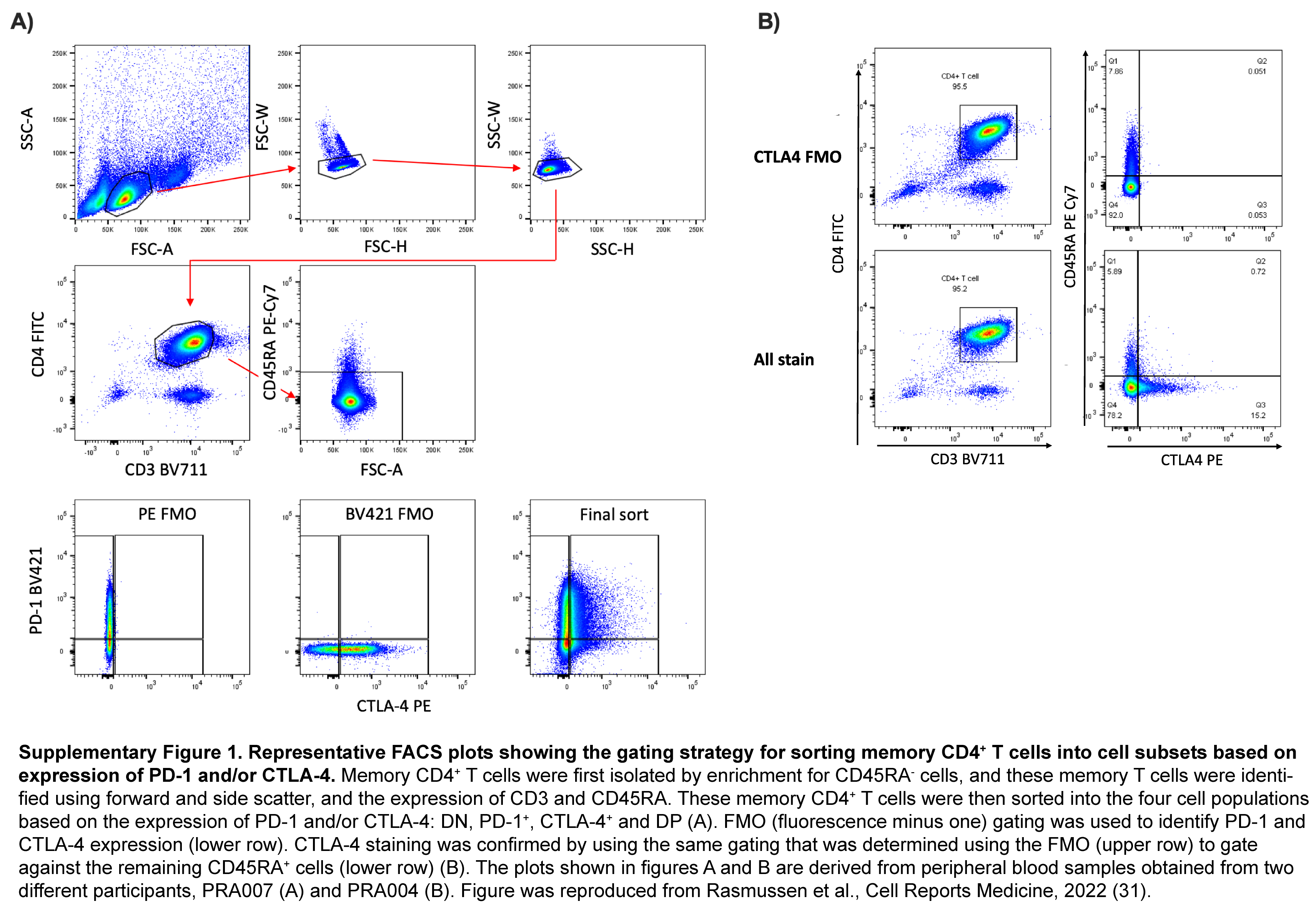

Supplement: Supplementary file 1 [file Image_1.tif]

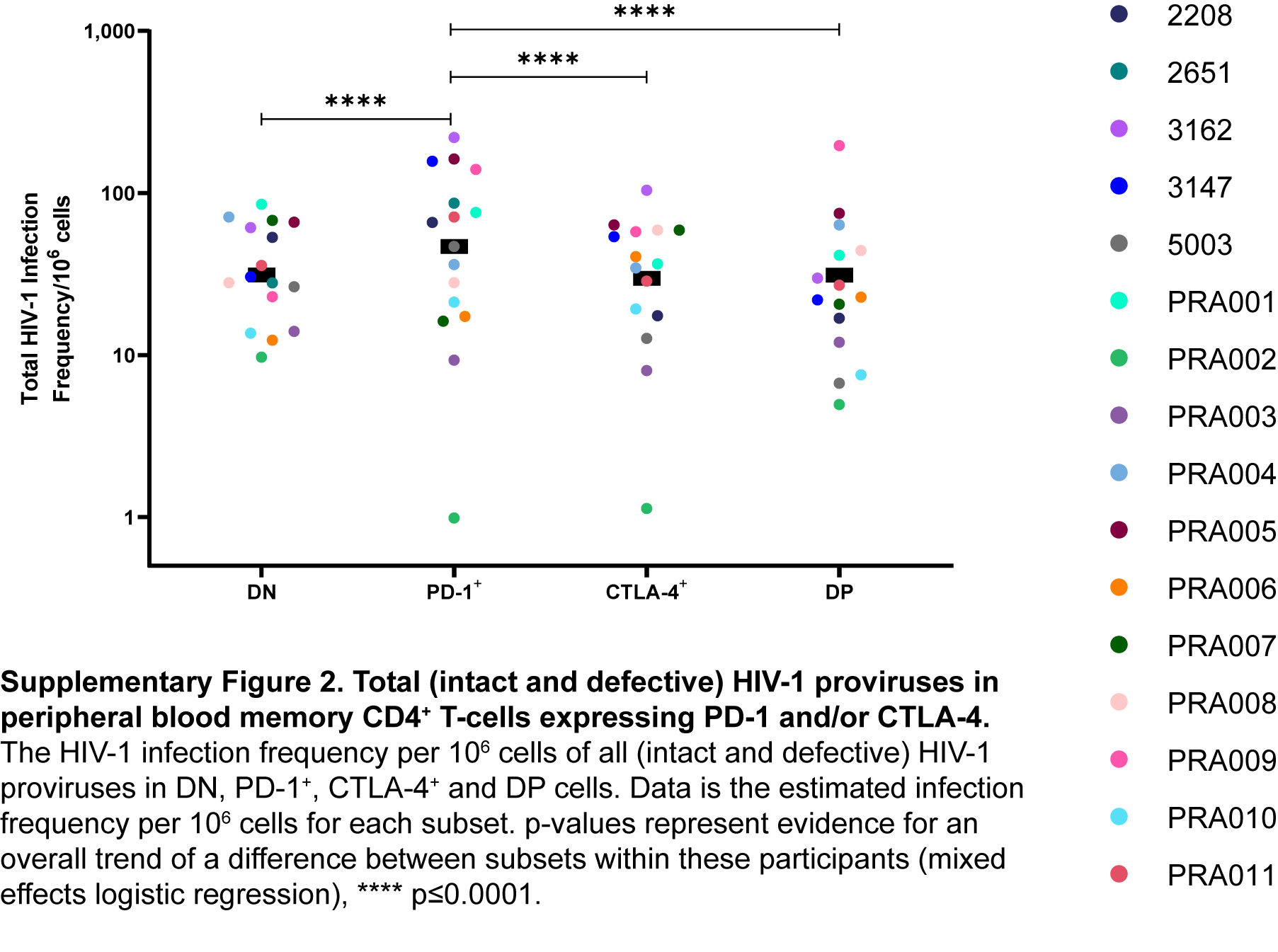

Supplement: Supplementary file 2 [file Image_2.tif]

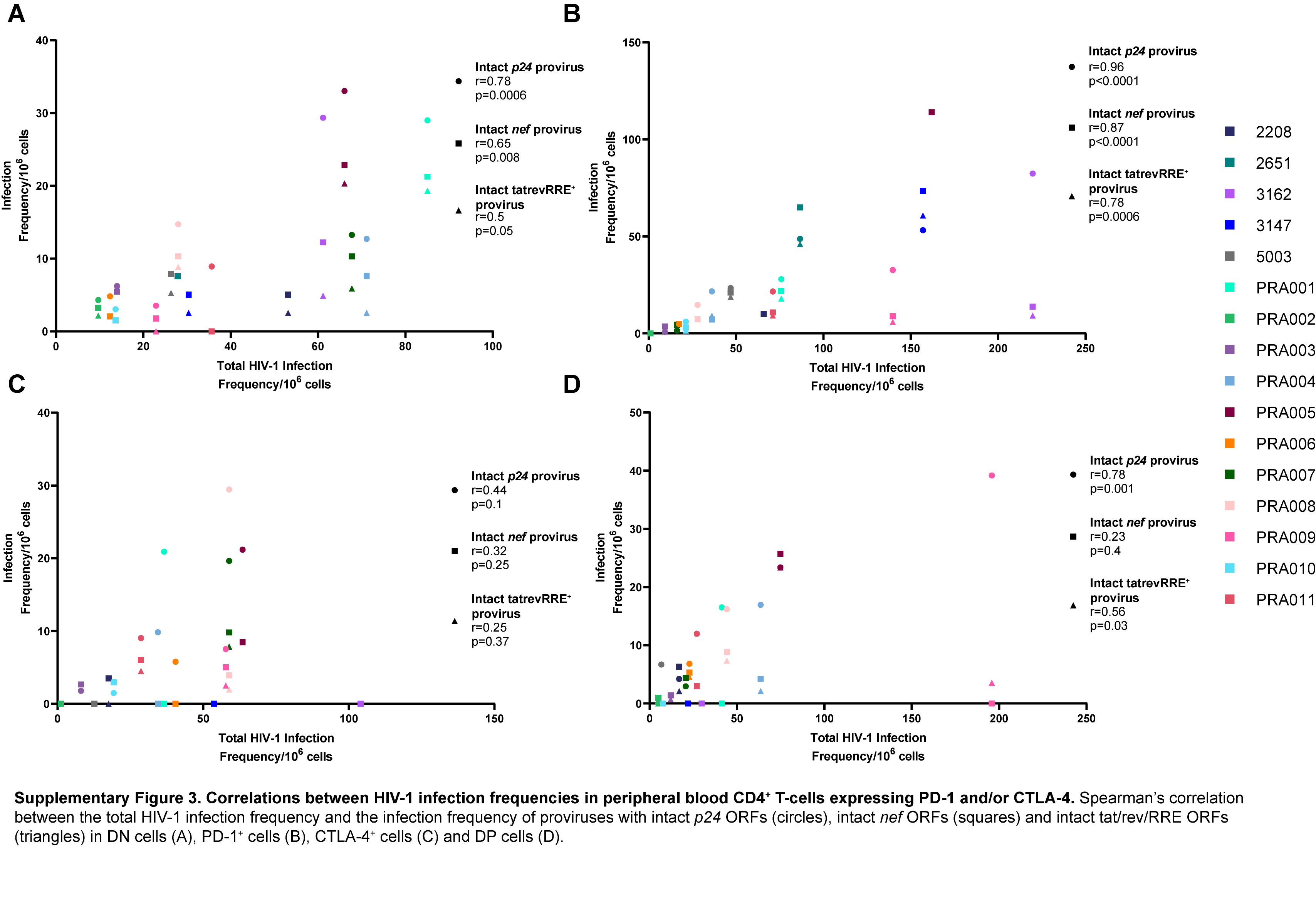

Supplement: Supplementary file 3 [file Image_3.tif]
