# Supplementary material for: Unequal distribution of genetically-intact HIV-1 proviruses in cells expressing the immune checkpoint markers PD-1 and/or CTLA-4
Source: Front Immunol. 2023 Jan 26;14:1064346. doi: 10.3389/fimmu.2023.1064346 (PMC9909745; doi:10.3389/fimmu.2023.1064346)
Supplement: Supplementary file 4 [file Table_1.docx]

| **PID^#^** | **Leuka-pheresis^#^** | **LN Biopsy^#^** | **Age (years)** | **Sex** | **Race** | **HIV diagnosis** | **Years since HIV diagnosis** | **HIV subtype** | **CD4 count (cells/µL)** | **CD4 % (%)** | **CD8 count (cells/µL)** | **CD8 % (%)** | **Nadir CD4 count (cells/µL)** | **ART regimen** | **VL (copies/ mL)** | **Peak VL (copies/ mL)** | **Duration VL <50 (years)** |
| --- | --- | --- | --- | --- | --- | --- | --- | --- | --- | --- | --- | --- | --- | --- | --- | --- | --- |
| **PRA001** | **Yes** | Yes | 64 | Male | Caucasian | 1985 | 31.7 | B | 403 | 24 | 1061 | 63 | 10 | ATV, TDF/FTC | <20 | 148,430 | 14.1 |
| **PRA002** | **Yes** | No | 48 | Male | Caucasian | 2006 | 10.9 | B | 1460 | 47 | 793 | 26 | 698 | ABC/3TC, EFV | <20 | N/A | N/A |
| **PRA003** | **Yes** | **Yes** | 49 | Male | Caucasian | 1997 | 20.3 | B | 833 | 31 | 767 | 29 | 218 | TDF/FTC, DRV, RTV | <20 | 78,300 | 11.5 |
| **PRA004** | **Yes** | No | 55 | Male | Caucasian | 1996 | 21.1 | B | 1036 | 40 | 1069 | 42 | 266 | TAF/FTC, DTG | <20 | 100,000 | 11.1 |
| **PRA005** | **Yes** | Yes | 49 | Male | Caucasian | 2003 | 13.7 | B | 388 | 28 | 717 | 51 | 168 | TAF/FTC, MVC | <20 | 147,000 | 12.0 |
| **PRA006** | **Yes** | **Yes** | 48 | Male | Caucasian | 2011 | 6.8 | B | 864 | 38 | 864 | 39 | 538 | EVG/TAF/FTC/COBI | <20 | 118,800 | 6.1 |
| **PRA007** | **Yes** | **Yes** | 47 | Male | Caucasian | 2001 | 16.4 | B | 705 | 32 | 1034 | 47 | 122 | DRV/COBI, TAF/FTC | <20 | 548,000 | 6.5 |
| **PRA008** | **Yes** | Yes | 38 | Male | Other (PNG) | 2006 | 11.2 | CRF01_ AE | 281 | 25 | 328 | 30 | 168 | EVG/TAF/FTC/COBI | <20 | 63,300 | 8.7 |
| **PRA009** | **Yes** | **Yes** | 49 | Male | Caucasian | 2010 | 7.5 | B | 474 | 25 | 1085 | 56 | 42 | EVG/TAF/FTC/COBI | <20 | 211,930 | 7.0 |
| **PRA010** | **Yes** | No | 48 | Male | Caucasian | 2000 | 17.6 | B | 484 | 28 | 895 | 52 | 411 | TAF, FTC, RPV | <20 | N/A | N/A |
| **PRA011** | **Yes** | Yes | 53 | Male | Caucasian | 2004 | 14.2 | B | 735 | 37 | 810 | 41 | 300 | ABC/3TC, EFV | <20 | 365,000 | 11.2 |
| **2208** | **Yes** | No | 66 | Male | Caucasian | 1984 | 34.2 | B | 466 | 31 | 546 | 36 | 54 | FTC/TAF, DRV/COBI | <40 | 50,000 | 9.8 |
| **2651** | **Yes** | No | 52 | Male | Caucasian | 2001 | 17.4 | B | 655 | 37 | 681 | 39 | 275 | ABC/DTG/3TC | <40 | 45,069 | 14.0 |
| **3162** | **Yes** | No | 56 | Male | Caucasian | 1987 | 31.0 | B | 586 | 37 | 471 | 30 | 200 | DRV, RTV, ABC/DTG/3TC | <40 | 171,000 | 11.5 |
| **3147** | **Yes** | No | 61 | Male | Hispanic/ Latino | 1993 | 25.3 | B | 837 | 44 | 522 | 27 | 4 | ABC/DTG/3TC | <40 | 119,870 | 11.0 |
| **5003** | **Yes** | No | 47 | Male | Hispanic/ Latino | 1993 | 25.1 | B | 279 | 25 | 385 | 35 | 56 | ATV, ABC/DTG/3TC | <40 | 171,000 | 6.7 |
| 1602 | Yes | No | 40 | Male | Native American | 2009 | 8.9 | B | 375 | 41 | 233 | 25 | 314 | BIC/FTC/TAF | <40 | 40,026 | 6.4 |
| 2013 | Yes | No | 70 | Male | Caucasian | 1986 | 32.7 | B | 524 | 40 | 395 | 30 | 13 | ABC/DTG/3TC | <40 | 70,070 | 9.9 |
| 2161 | Yes | No | 69 | Male | Caucasian | 1985 | 33.3 | B | 800 | 42 | 647 | 34 | 98 | 3TC, DRV, RTV, DTG | <40 | 80,410 | 7.0 |
| 2253 | Yes | No | 67 | Male | Caucasian | 2000 | 18.4 | B | 463 | 31 | 462 | 31 | 380 | BIC/FTC/TAF | <40 | 376,658 | 4.2 |
| 2467 | Yes | No | 46 | Male | Hispanic/ Latino | 2006 | 11.9 | B | 429 | 43 | 316 | 32 | 324 | RPV/TAF/FTC | <40 | 47,100 | 10.4 |
| Mean - full cohort (sd) | N/A | N/A | 53 (9.3) | N/A | N/A | N/A | 19.5 (9.0) | N/A | 623 (284) | 35 (7.1) | 671 (270) | 38 (10.6) | 222 (183) | N/A | N/A | 155,366 (135,841) | 9.4 (2.8) |
| Mean - sub study (sd) | N/A | N/A | 52 (6.9) | N/A | N/A | N/A | 19.0 (8.3) | N/A | 655 (299) | 33 (6.9) | 752 (240) | 40 (10.7) | 221 (189) | N/A | N/A | 166,979 (131,847) | 10.1 (2.6) |

**Supplementary Table 1. Clinical characteristics of Participants in full cohort described in Rasmussen et al. (2022)**

^#^ Bolded PIDs and sample types were included in the sub study

ART: antiretroviral therapy; VL: viral load; LN: lymph node; N/A: not applicable
