# Supplementary material for: Unequal distribution of genetically-intact HIV-1 proviruses in cells expressing the immune checkpoint markers PD-1 and/or CTLA-4
Source: Front Immunol. 2023 Jan 26;14:1064346. doi: 10.3389/fimmu.2023.1064346 (PMC9909745; doi:10.3389/fimmu.2023.1064346)
Supplement: Supplementary file 5 [file Table_2.docx]

**Supplementary Table 2. Calculated p-values indicating differences in all properties of HIV-1 proviruses compared between subsets or participant effect modification**

| **Comparison** | **Measurement** | **Anatomic location** | **Subsets compared** | **Difference between subsets in this data (p-value)** | **Direction** | **Participant effect modification (p-value)** |
| --- | --- | --- | --- | --- | --- | --- |
| Cells HIV-1^+^ positive | Infection frequency per 10^6^ cells | PB | DN vs PD-1^+^ | <0.00001 | PD-1^+^>DN | <0.00001 |
|  |  |  | DN vs CTLA-4^+^ | 0.85 |  | <0.00001 |
|  |  |  | DN vs DP | 0.78 |  | <0.00001 |
|  |  |  | PD-1^+^ vs CTLA-4^+^ | <0.00001 | PD-1^+^>CTLA-4^+^ | <0.00001 |
|  |  |  | PD-1^+^ vs DP | 0.0001 | PD-1^+^>DP | <0.00001 |
|  |  |  | CTLA-4^+^ vs DP | 0.86 |  | <0.00001 |
|  |  | LN | DN vs PD-1^+^ | 0.61 |  | 0.2 |
|  |  |  | DN vs CTLA-4^+^ | 0.006 | DN>CTLA-4^+^ | 0.05 |
|  |  |  | DN vs DP | 0.19 |  | <0.00001 |
|  |  |  | PD-1^+^ vs CTLA-4^+^ | 0.001 | PD-1^+^>CTLA-4^+^ | 0.03 |
|  |  |  | PD-1^+^ vs DP | 0.16* |  | <0.00001 |
|  |  |  | CTLA-4^+^ vs DP | 0.0002 | DP>CTLA-4^+^ | 0.6 |
| Genetically-Intact | Infection frequency per 10^6^ cells | PB | DN vs PD-1^+^ | 0.72 |  | 0.009 |
|  |  |  | DN vs CTLA-4^+^ | 0.01 | DN>CTLA-4^+^ | 0.34 |
|  |  |  | DN vs DP | 0.03 | DN>DP | 0.3 |
|  |  |  | PD-1^+^ vs CTLA-4^+^ | 0.04 | PD-1^+^>CTLA-4^+^ | 0.19 |
|  |  |  | PD-1^+^ vs DP | 0.2 |  | 0.21 |
|  |  |  | CTLA-4^+^ vs DP | 0.92* |  | 0.98 |
|  |  | LN | DN vs PD-1^+^ | 0.06* |  | 1 |
|  |  |  | DN vs CTLA-4^+^ | 1* |  | 1 |
|  |  |  | DN vs DP | 0.59* |  | 1 |
|  |  |  | PD-1^+^ vs CTLA-4^+^ | 0.12* |  | 1 |
|  |  |  | PD-1^+^ vs DP | 0.13* |  | 0.69 |
|  |  |  | CTLA-4^+^ vs DP | 0.41* |  | 1 |
| Genetically-Intact | Proportion proviruses | PB | DN vs PD-1^+^ | 0.15 |  | 0.47 |
|  |  |  | DN vs CTLA-4^+^ | 0.02 | DN>CTLA-4^+^ | 0.52 |
|  |  |  | DN vs DP | 0.11 |  | 0.6 |
|  |  |  | PD-1^+^ vs CTLA-4^+^ | 0.31 |  | 0.65 |
|  |  |  | PD-1^+^ vs DP | 0.62 |  | 0.43 |
|  |  |  | CTLA-4^+^ vs DP | 0.56 |  | 0.95 |
|  |  | LN | DN vs PD-1^+^ | 0.09* |  | 1 |
|  |  |  | DN vs CTLA-4^+^ | 1* |  | N/A |
|  |  |  | DN vs DP | 1* |  | 1 |
|  |  |  | PD-1^+^ vs CTLA-4^+^ | 1* |  | N/A |
|  |  |  | PD-1^+^ vs DP | 0.65* |  | 0.98 |
|  |  |  | CTLA-4^+^ vs DP | 1* |  | N/A |
| Intact *p24* ORF | Infection frequency per 10^6^ cells | PB | DN vs PD-1^+^ | <0.00001 | PD-1^+^>DN | <0.00001 |
|  |  |  | DN vs CTLA-4^+^ | 0.29 |  | 0.06 |
|  |  |  | DN vs DP | 0.33 |  | 0.0003 |
|  |  |  | PD-1^+^ vs CTLA-4^+^ | <0.00001 | PD-1^+^>CTLA-4^+^ | <0.00001 |
|  |  |  | PD-1^+^ vs DP | <0.00001 | PD-1^+^>DP | 0.0005 |
|  |  |  | CTLA-4^+^ vs DP | 0.88* |  | 0.05 |
|  |  | LN | DN vs PD-1^+^ | 0.58 |  | 0.24 |
|  |  |  | DN vs CTLA-4^+^ | 0.28 |  | 0.14 |
|  |  |  | DN vs DP | 0.53* |  | 0.001 |
|  |  |  | PD-1^+^ vs CTLA-4^+^ | 0.09 |  | 0.28 |
|  |  |  | PD-1^+^ vs DP | 0.39* |  | 0.02 |
|  |  |  | CTLA-4^+^ vs DP | 0.19 |  | 0.62 |
| Intact *p24* ORF | Proportion proviruses | PB | DN vs PD-1^+^ | 0.11 |  | 0.01 |
|  |  |  | DN vs CTLA-4^+^ | 0.1 |  | 0.07 |
|  |  |  | DN vs DP | 0.26 |  | 0.21 |
|  |  |  | PD-1^+^ vs CTLA-4^+^ | 0.0001 | PD-1^+^>CTLA-4^+^ | 0.002 |
|  |  |  | PD-1^+^ vs DP | 0.003 | PD-1^+^>DP | 0.2 |
|  |  |  | CTLA-4^+^ vs DP | 0.65 |  | 0.69 |
|  |  | LN | DN vs PD-1^+^ | 0.62* |  | 0.01 |
|  |  |  | DN vs CTLA-4^+^ | 0.19* |  | N/A |
|  |  |  | DN vs DP | 1* |  | 0.13 |
|  |  |  | PD-1^+^ vs CTLA-4^+^ | 0.14* |  | N/A |
|  |  |  | PD-1^+^ vs DP | 0.88* |  | 0.14 |
|  |  |  | CTLA-4^+^ vs DP | 0.71* |  | N/A |
| Intact *p24* ORF with *gag* start codon correct | Infection frequency per 10^6^ cells | PB | DN vs PD-1^+^ | 0.0004 | PD-1^+^>DN | <0.00001 |
|  |  |  | DN vs CTLA-4^+^ | 0.79 |  | 0.02 |
|  |  |  | DN vs DP | 0.49 |  | 0.0001 |
|  |  |  | PD-1^+^ vs CTLA-4^+^ | 0.006 | PD-1^+^>CTLA-4^+^ | <0.00001 |
|  |  |  | PD-1^+^ vs DP | 0.02 | PD-1^+^>DP | 0.007 |
|  |  |  | CTLA-4^+^ vs DP | 0.47 |  | 0.02 |
|  |  | LN | DN vs PD-1^+^ | 0.63 |  | 0.26 |
|  |  |  | DN vs CTLA-4^+^ | 0.07* |  | 1 |
|  |  |  | DN vs DP | 0.53* |  | 0.001 |
|  |  |  | PD-1^+^ vs CTLA-4^+^ | 0.004* | PD-1^+^>CTLA-4^+^ | 1 |
|  |  |  | PD-1^+^ vs DP | 0.52* |  | 0.02 |
|  |  |  | CTLA-4^+^ vs DP | 0.01* | DP>CTLA-4^+^ | 1 |
| Intact *p24* ORF with *gag* start codon correct | Proportion proviruses | PB | DN vs PD-1^+^ | 0.81 |  | 0.07 |
|  |  |  | DN vs CTLA-4^+^ | 0.54 |  | 0.05 |
|  |  |  | DN vs DP | 0.4 |  | 0.26 |
|  |  |  | PD-1^+^ vs CTLA-4^+^ | 0.28 |  | 0.0004 |
|  |  |  | PD-1^+^ vs DP | 0.49 |  | 0.77 |
|  |  |  | CTLA-4^+^ vs DP | 0.8 |  | 0.43 |
|  |  | LN | DN vs PD-1^+^ | 0.62* |  | 0.01 |
|  |  |  | DN vs CTLA-4^+^ | 1* |  | N/A |
|  |  |  | DN vs DP | 1* |  | 0.13 |
|  |  |  | PD-1^+^ vs CTLA-4^+^ | 0.01* | PD-1^+^>CTLA-4^+^ | N/A |
|  |  |  | PD-1^+^ vs DP | 0.92* |  | 0.12 |
|  |  |  | CTLA-4^+^ vs DP | 0.28* |  | N/A |
| Intact *nef* ORF | Infection frequency per 10^6^ cells | PB | DN vs PD-1^+^ | <0.00001 | PD-1^+^>DN | <0.00001 |
|  |  |  | DN vs CTLA-4^+^ | 0.002 | DN>CTLA-4^+^ | 0.02 |
|  |  |  | DN vs DP | 0.07 |  | 0.12 |
|  |  |  | PD-1^+^ vs CTLA-4^+^ | <0.00001 | PD-1^+^>CTLA-4^+^ | 0.0001 |
|  |  |  | PD-1^+^ vs DP | <0.00001 | PD-1^+^>DP | 0.01 |
|  |  |  | CTLA-4^+^ vs DP | 0.25 |  | 0.16 |
|  |  | LN | DN vs PD-1^+^ | 0.23 |  | 0.18 |
|  |  |  | DN vs CTLA-4^+^ | 0.85 |  | 0.8 |
|  |  |  | DN vs DP | 0.35* |  | 0.64 |
|  |  |  | PD-1^+^ vs CTLA-4^+^ | 0.13* |  | 0.04 |
|  |  |  | PD-1^+^ vs DP | 0.006 | PD-1^+^>DP | 0.74 |
|  |  |  | CTLA-4^+^ vs DP | 0.98* |  | 0.52 |
| Intact *nef* ORF | Proportion proviruses | PB | DN vs PD-1^+^ | 0.002 | PD-1^+^>DN | 0.007 |
|  |  |  | DN vs CTLA-4^+^ | 0.0006 | DN>CTLA-4^+^ | 0.006 |
|  |  |  | DN vs DP | 0.31 |  | 0.17 |
|  |  |  | PD-1^+^ vs CTLA-4^+^ | <0.00001 | PD-1^+^>CTLA-4^+^ | 0.004 |
|  |  |  | PD-1^+^ vs DP | 0.0001 | PD-1^+^>DP | 0.43 |
|  |  |  | CTLA-4^+^ vs DP | 0.03 | DP>CTLA-4^+^ | 0.14 |
|  |  | LN | DN vs PD-1^+^ | 0.41* |  | 0.05 |
|  |  |  | DN vs CTLA-4^+^ | 1* |  | N/A |
|  |  |  | DN vs DP | 0.12* |  | 1 |
|  |  |  | PD-1^+^ vs CTLA-4^+^ | 0.14* |  | N/A |
|  |  |  | PD-1^+^ vs DP | 0.03 | PD-1^+^>DP | 0.97 |
|  |  |  | CTLA-4^+^ vs DP | 0.14* |  | N/A |
| Intact *tat* ORF | Infection frequency per 10^6^ cells | PB | DN vs PD-1^+^ | <0.00001 | PD-1^+^>DN | <0.00001 |
|  |  |  | DN vs CTLA-4^+^ | 0.07 |  | 0.11 |
|  |  |  | DN vs DP | 0.22 |  | 0.04 |
|  |  |  | PD-1^+^ vs CTLA-4^+^ | <0.00001 | PD-1^+^>CTLA-4^+^ | <0.00001 |
|  |  |  | PD-1^+^ vs DP | <0.00001 | PD-1^+^>DP | 0.02 |
|  |  |  | CTLA-4^+^ vs DP | 0.72 |  | 0.26 |
|  |  | LN | DN vs PD-1^+^ | 0.08* |  | 0.08 |
|  |  |  | DN vs CTLA-4^+^ | 0.95 |  | 1 |
|  |  |  | DN vs DP | 0.59 |  | 0.99 |
|  |  |  | PD-1^+^ vs CTLA-4^+^ | 0.18* |  | 0.05 |
|  |  |  | PD-1^+^ vs DP | 0.02* | PD-1^+^>DP | 0.03 |
|  |  |  | CTLA-4^+^ vs DP | 0.67 |  | 0.88 |
| Intact *tat* ORF | Proportion proviruses | PB | DN vs PD-1^+^ | 0.0003 | PD-1^+^>DN | 0.01 |
|  |  |  | DN vs CTLA-4^+^ | 0.05 | DN>CTLA-4^+^ | 0.16 |
|  |  |  | DN vs DP | 0.55 |  | 0.53 |
|  |  |  | PD-1^+^ vs CTLA-4^+^ | <0.00001 | PD-1^+^>CTLA-4^+^ | 0.05 |
|  |  |  | PD-1^+^ vs DP | 0.0001 | PD-1^+^>DP | 0.6 |
|  |  |  | CTLA-4^+^ vs DP | 0.12 |  | 0.95 |
|  |  | LN | DN vs PD-1^+^ | 0.16* |  | 0.01 |
|  |  |  | DN vs CTLA-4^+^ | 0.66 |  | N/A |
|  |  |  | DN vs DP | 0.93 |  | 1 |
|  |  |  | PD-1^+^ vs CTLA-4^+^ | 0.14* |  | N/A |
|  |  |  | PD-1^+^ vs DP | 0.2* |  | 0.29 |
|  |  |  | CTLA-4^+^ vs DP | 0.71* |  | N/A |
| Intact *rev* ORF | Infection frequency per 10^6^ cells | PB | DN vs PD-1^+^ | <0.00001 | PD-1^+^>DN | 0.001 |
|  |  |  | DN vs CTLA-4^+^ | 0.007 | DN>CTLA-4^+^ | 0.03 |
|  |  |  | DN vs DP | 0.3 |  | 0.05 |
|  |  |  | PD-1^+^ vs CTLA-4^+^ | <0.00001 | PD-1^+^>CTLA-4^+^ | 0.0002 |
|  |  |  | PD-1^+^ vs DP | <0.00001 | PD-1^+^>DP | 0.03 |
|  |  |  | CTLA-4^+^ vs DP | 0.18 |  | 0.35 |
|  |  | LN | DN vs PD-1^+^ | 0.08* |  | 0.08 |
|  |  |  | DN vs CTLA-4^+^ | 0.95 |  | 1 |
|  |  |  | DN vs DP | 0.81* |  | 0.83 |
|  |  |  | PD-1^+^ vs CTLA-4^+^ | 0.18* |  | 0.05 |
|  |  |  | PD-1^+^ vs DP | 0.01 | PD-1^+^>DP | 0.7 |
|  |  |  | CTLA-4^+^ vs DP | 0.98* |  | 0.52 |
| Intact *rev* ORF | Proportion proviruses | PB | DN vs PD-1^+^ | 0.0003 | PD-1^+^>DN | 0.08 |
|  |  |  | DN vs CTLA-4^+^ | 0.005 | DN>CTLA-4^+^ | 0.01 |
|  |  |  | DN vs DP | 0.52 |  | 0.36 |
|  |  |  | PD-1^+^ vs CTLA-4^+^ | <0.00001 | PD-1^+^>CTLA-4^+^ | 0.004 |
|  |  |  | PD-1^+^ vs DP | <0.00001 | PD-1^+^>DP | 0.59 |
|  |  |  | CTLA-4^+^ vs DP | 0.03 | DP>CTLA-4^+^ | 0.19 |
|  |  | LN | DN vs PD-1^+^ | 0.16* |  | 0.01 |
|  |  |  | DN vs CTLA-4^+^ | 0.66 |  | N/A |
|  |  |  | DN vs DP | 0.14* |  | 1 |
|  |  |  | PD-1^+^ vs CTLA-4^+^ | 0.14* |  | N/A |
|  |  |  | PD-1^+^ vs DP | 0.06* |  | 0.95 |
|  |  |  | CTLA-4^+^ vs DP | 0.14* |  | N/A |
| Intact tat/rev/RRE provirus | Infection frequency per 10^6^ cells | PB | DN vs PD-1^+^ | <0.00001 | PD-1^+^>DN | 0.0001 |
|  |  |  | DN vs CTLA-4^+^ | 0.008* | DN>CTLA-4^+^ | 0.07 |
|  |  |  | DN vs DP | 0.24* |  | 0.09 |
|  |  |  | PD-1^+^ vs CTLA-4^+^ | <0.00001 | PD-1^+^>CTLA-4^+^ | 0.0005 |
|  |  |  | PD-1^+^ vs DP | <0.00001 | PD-1^+^>DP | 0.11 |
|  |  |  | CTLA-4^+^ vs DP | 0.22 |  | 0.4 |
|  |  | LN | DN vs PD-1^+^ | 0.08* |  | 0.08 |
|  |  |  | DN vs CTLA-4^+^ | 0.95 |  | 1 |
|  |  |  | DN vs DP | 0.81* |  | 0.83 |
|  |  |  | PD-1^+^ vs CTLA-4^+^ | 0.18* |  | 0.05 |
|  |  |  | PD-1^+^ vs DP | 0.01 | PD-1^+^>DP | 0.7 |
|  |  |  | CTLA-4^+^ vs DP | 0.98* |  | 0.52 |
| Intact tat/rev/RRE provirus | Proportion proviruses | PB | DN vs PD-1^+^ | 0.0001 | PD-1^+^>DN | 0.02 |
|  |  |  | DN vs CTLA-4^+^ | 0.006 | DN>CTLA-4^+^ | 0.03 |
|  |  |  | DN vs DP | 0.46 |  | 0.26 |
|  |  |  | PD-1^+^ vs CTLA-4^+^ | <0.00001 | PD-1^+^>CTLA-4^+^ | 0.04 |
|  |  |  | PD-1^+^ vs DP | <0.00001 | PD-1^+^>DP | 0.69 |
|  |  |  | CTLA-4^+^ vs DP | 0.03 | DP>CTLA-4^+^ | 0.48 |
|  |  | LN | DN vs PD-1^+^ | 0.16* |  | 0.01 |
|  |  |  | DN vs CTLA-4^+^ | 0.66 |  | N/A |
|  |  |  | DN vs DP | 0.14* |  | 1 |
|  |  |  | PD-1^+^ vs CTLA-4^+^ | 0.14* |  | N/A |
|  |  |  | PD-1^+^ vs DP | 0.06* |  | 0.95 |
|  |  |  | CTLA-4^+^ vs DP | 0.14* |  | N/A |
| CCR5-tropic provirus | Proportion proviruses | PB | DN vs PD-1^+^ | <0.00001 | PD-1^+^>DN | 0.002 |
|  |  |  | DN vs CTLA-4^+^ | 0.04 | DN>CTLA-4^+^ | 0.03 |
|  |  |  | DN vs DP | 0.88 |  | 0.59 |
|  |  |  | PD-1^+^ vs CTLA-4^+^ | <0.00001 | PD-1^+^>CTLA-4^+^ | 0.03 |
|  |  |  | PD-1^+^ vs DP | <0.00001 | PD-1^+^>DP | 0.21 |
|  |  |  | CTLA-4^+^ vs DP | 0.15 |  | 0.21 |
|  |  | LN | DN vs PD-1^+^ | 0.27* |  | 0.05 |
|  |  |  | DN vs CTLA-4^+^ | 0.19* |  | N/A |
|  |  |  | DN vs DP | 0.92* |  | 0.51 |
|  |  |  | PD-1^+^ vs CTLA-4^+^ | 0.01* | CTLA-4^+^>PD-1^+^ | N/A |
|  |  |  | PD-1^+^ vs DP | 0.49* |  | 0.03 |
|  |  |  | CTLA-4^+^ vs DP | 0.28* |  | N/A |
| CXCR4-tropic provirus | Proportion proviruses | PB | DN vs PD-1^+^ | 0.03 | DN>PD-1^+^ | 0.29 |
|  |  |  | DN vs CTLA-4^+^ | 0.72 |  | 0.87 |
|  |  |  | DN vs DP | 0.08 |  | 0.63 |
|  |  |  | PD-1^+^ vs CTLA-4^+^ | 0.12 |  | 0.46 |
|  |  |  | PD-1^+^ vs DP | 0.13 |  | 0.49 |
|  |  |  | CTLA-4^+^ vs DP | 0.27 |  | 0.44 |
|  |  | LN | DN vs PD-1^+^ | 0.23* |  | 1 |
|  |  |  | DN vs CTLA-4^+^ | 1* |  | N/A |
|  |  |  | DN vs DP | 1* |  | 1 |
|  |  |  | PD-1^+^ vs CTLA-4^+^ | 1* |  | N/A |
|  |  |  | PD-1^+^ vs DP | 0.71* |  | 1 |
|  |  |  | CTLA-4^+^ vs DP | 1* |  | N/A |
| Sequences part of an EIS | Proportion proviruses | PB | DN vs PD-1^+^ | 0.001 | PD-1^+^>DN | <0.00001 |
|  |  |  | DN vs CTLA-4^+^ | 0.24 |  | 0.0001 |
|  |  |  | DN vs DP | 0.84 |  | 0.03 |
|  |  |  | PD-1^+^ vs CTLA-4^+^ | 0.02 | PD-1^+^>CTLA-4^+^ | <0.00001 |
|  |  |  | PD-1^+^ vs DP | 0.049 | PD-1^+^>DP | 0.01 |
|  |  |  | CTLA-4^+^ vs DP | 0.23 |  | 0.008 |
|  |  | LN | DN vs PD-1^+^ | 0.002* | DN>PD-1^+^ | 1 |
|  |  |  | DN vs CTLA-4^+^ | 1* |  | N/A |
|  |  |  | DN vs DP | 0.3* |  | 1 |
|  |  |  | PD-1^+^ vs CTLA-4^+^ | 1* |  | N/A |
|  |  |  | PD-1^+^ vs DP | 0.004* | DP>PD-1^+^ | 1 |
|  |  |  | CTLA-4^+^ vs DP | 1* |  | N/A |
| Sequences length >8800bp | Proportion proviruses | PB | DN vs PD-1^+^ | 0.06 |  | 0.005 |
|  |  |  | DN vs CTLA-4^+^ | 0.14 |  | 0.47 |
|  |  |  | DN vs DP | 0.2 |  | 0.22 |
|  |  |  | PD-1^+^ vs CTLA-4^+^ | 0.0002 | PD-1^+^>CTLA-4^+^ | 0.0001 |
|  |  |  | PD-1^+^ vs DP | 0.004 | PD-1^+^>DP | 0.01 |
|  |  |  | CTLA-4^+^ vs DP | 0.95 |  | 0.35 |
|  |  | LN | DN vs PD-1^+^ | 0.06* |  | 1 |
|  |  |  | DN vs CTLA-4^+^ | 0.02* | CTLA-4^+^>DN | N/A |
|  |  |  | DN vs DP | 0.51* |  | 0.34 |
|  |  |  | PD-1^+^ vs CTLA-4^+^ | 0.01* | CTLA-4^+^>PD-1^+^ | N/A |
|  |  |  | PD-1^+^ vs DP | 0.81* |  | 0.14 |
|  |  |  | CTLA-4^+^ vs DP | 0.09* |  | N/A |
| Proviruses length >8800bp with MSD intact | Proportion proviruses | PB | DN vs PD-1^+^ | 0.001 | DN>PD-1^+^ | 0.22 |
|  |  |  | DN vs CTLA-4^+^ | 0.06 |  | 0.67 |
|  |  |  | DN vs DP | 0.82 |  | 0.43 |
|  |  |  | PD-1^+^ vs CTLA-4^+^ | <0.00001 | CTLA-4^+^>PD-1^+^ | 1 |
|  |  |  | PD-1^+^ vs DP | 0.003 | DP>PD-1^+^ | 0.6 |
|  |  |  | CTLA-4^+^ vs DP | 0.16 |  | 0.98 |
|  |  | LN | DN vs PD-1^+^ | 1* |  | N/A |
|  |  |  | DN vs CTLA-4^+^ | N/A |  | N/A |
|  |  |  | DN vs DP | N/A |  | N/A |
|  |  |  | PD-1^+^ vs CTLA-4^+^ | Error |  | N/A |
|  |  |  | PD-1^+^ vs DP | 1* |  | N/A |
|  |  |  | CTLA-4^+^ vs DP | 1* |  | N/A |
| Proviruses with an inversion | Proportion | PB | DN vs PD-1^+^ | 0.2* |  | 0.55 |
|  |  |  | DN vs CTLA-4^+^ | 0.83 |  | 0.89 |
|  |  |  | DN vs DP | 0.09 |  | 0.3 |
|  |  |  | PD-1^+^ vs CTLA-4^+^ | 0.46 |  | 0.95 |
|  |  |  | PD-1^+^ vs DP | 0.32 |  | 0.61 |
|  |  |  | CTLA-4^+^ vs DP | 0.1 |  | 0.78 |
|  |  | LN | DN vs PD-1^+^ | 0.23* |  | 1 |
|  |  |  | DN vs CTLA-4^+^ | 1* |  | N/A |
|  |  |  | DN vs DP | 0.03* | DP>DN | 1 |
|  |  |  | PD-1^+^ vs CTLA-4^+^ | 1* |  | N/A |
|  |  |  | PD-1^+^ vs DP | 0.009 | DP>PD-1^+^ | 1 |
|  |  |  | CTLA-4^+^ vs DP | 0.93 |  | N/A |
| Proviruses with a deletion | Proportion | PB | DN vs PD-1^+^ | 0.03 | DN>PD-1^+^ | 0.0002 |
|  |  |  | DN vs CTLA-4^+^ | 0.23 |  | 0.36 |
|  |  |  | DN vs DP | 0.91 |  | 0.02 |
|  |  |  | PD-1^+^ vs CTLA-4^+^ | 0.0001 | CTLA-4^+^>PD-1^+^ | <0.00001 |
|  |  |  | PD-1^+^ vs DP | 0.04 | DP>PD-1^+^ | 0.008 |
|  |  |  | CTLA-4^+^ vs DP | 0.33 |  | 0.19 |
|  |  | LN | DN vs PD-1^+^ | 0.18* |  | 1 |
|  |  |  | DN vs CTLA-4^+^ | 0.02* | DN>CTLA-4^+^ | N/A |
|  |  |  | DN vs DP | 0.03* |  | 1 |
|  |  |  | PD-1^+^ vs CTLA-4^+^ | 0.01* | PD-1^+^>CTLA-4^+^ | N/A |
|  |  |  | PD-1^+^ vs DP | 0.67* |  | 0.09 |
|  |  |  | CTLA-4^+^ vs DP | 0.09* |  | N/A |
| Hypermutated proviruses | Proportion | PB | DN vs PD-1^+^ | 0.45* |  | 0.39 |
|  |  |  | DN vs CTLA-4^+^ | 0.44 |  | 0.72 |
|  |  |  | DN vs DP | 0.91 |  | 0.14 |
|  |  |  | PD-1^+^ vs CTLA-4^+^ | 0.14* |  | 0.23 |
|  |  |  | PD-1^+^ vs DP | 0.37 |  | 0.046 |
|  |  |  | CTLA-4^+^ vs DP | 0.33 |  | 0.33 |
|  |  | LN | DN vs PD-1^+^ | 0.43 |  | 1 |
|  |  |  | DN vs CTLA-4^+^ | 0.19* |  | N/A |
|  |  |  | DN vs DP | 0.51* |  | 0.34 |
|  |  |  | PD-1^+^ vs CTLA-4^+^ | 0.22 |  | N/A |
|  |  |  | PD-1^+^ vs DP | 0.47* |  | 0.07 |
|  |  |  | CTLA-4^+^ vs DP | 0.71* |  | N/A |
| Proviruses with a premature stop codon in an ORF | Proportion | PB | DN vs PD-1^+^ | 0.6 |  | 0.97 |
|  |  |  | DN vs CTLA-4^+^ | 1* |  | 1 |
|  |  |  | DN vs DP | 1* |  | 1 |
|  |  |  | PD-1^+^ vs CTLA-4^+^ | 0.04* | PD-1^+^>CTLA-4^+^ | 1 |
|  |  |  | PD-1^+^ vs DP | 0.06* |  | 1 |
|  |  |  | CTLA-4^+^ vs DP | 1* |  | 1 |
|  |  | LN | DN vs PD-1^+^ | 1* |  | 1 |
|  |  |  | DN vs CTLA-4^+^ | 1* |  | N/A |
|  |  |  | DN vs DP | 1* |  | 1 |
|  |  |  | PD-1^+^ vs CTLA-4^+^ | 1* |  | N/A |
|  |  |  | PD-1^+^ vs DP | 1* |  | 1 |
|  |  |  | CTLA-4^+^ vs DP | 1* |  | N/A |
| Proviruses with a frameshift mutation in an ORF | Proportion | PB | DN vs PD-1^+^ | 0.11 |  | 0.98 |
|  |  |  | DN vs CTLA-4^+^ | 0.16* |  | 1 |
|  |  |  | DN vs DP | 0.16* |  | 1 |
|  |  |  | PD-1^+^ vs CTLA-4^+^ | 0.0001* |  | 1 |
|  |  |  | PD-1^+^ vs DP | 0.32* |  | 1 |
|  |  |  | CTLA-4^+^ vs DP | 1* |  | 1 |
|  |  | LN | DN vs PD-1^+^ | 1* |  | 1 |
|  |  |  | DN vs CTLA-4^+^ | 0.19* |  | N/A |
|  |  |  | DN vs DP | 1* |  | 1 |
|  |  |  | PD-1^+^ vs CTLA-4^+^ | 0.14* |  | N/A |
|  |  |  | PD-1^+^ vs DP | 0.29* |  | 1 |
|  |  |  | CTLA-4^+^ vs DP | 0.14* |  | N/A |
| Proviruses with a *cis*-acting defect | Proportion | PB | DN vs PD-1^+^ | <0.00001 | PD-1^+^>DN | 0.2 |
|  |  |  | DN vs CTLA-4^+^ | 0.08 |  | 0.99 |
|  |  |  | DN vs DP | 0.98 |  | 0.97 |
|  |  |  | PD-1^+^ vs CTLA-4^+^ | <0.00001 | PD-1^+^>CTLA-4^+^ | 1 |
|  |  |  | PD-1^+^ vs DP | <0.00001 | PD-1^+^>DP | 0.99 |
|  |  |  | CTLA-4^+^ vs DP | 0.14 |  | 1 |
|  |  | LN | DN vs PD-1^+^ | 1* |  | 1 |
|  |  |  | DN vs CTLA-4^+^ | 1* |  | N/A |
|  |  |  | DN vs DP | 1* |  | 1 |
|  |  |  | PD-1^+^ vs CTLA-4^+^ | 1* |  | N/A |
|  |  |  | PD-1^+^ vs DP | 0.35* |  | 1 |
|  |  |  | CTLA-4^+^ vs DP | 1* |  | N/A |
